# Towards Sustainable Energy‐Saving Hydrogen Production: Recent Advances in Tungsten‐Based Urea Oxidation Electrocatalysts

**DOI:** 10.1002/smll.75641

**Published:** 2026-09-09

**Authors:** Yi Zeng, Shun Lu, Yuan Wang, Saddick Donkor, Donna Cairns, Xingqun Zheng, Qingsong Hua, Ben Bin Xu

**Affiliations:** ^1^ School of Physics and Astronomy Beijing Normal University Beijing China; ^2^ Chongqing Institute of Green and Intelligent Technology Chinese Academy of Sciences Chongqing China; ^3^ School of Engineering Physics and Mathematics Faculty of Science and Environment Northumbria University Newcastle upon Tyne UK; ^4^ School of Safety Science and Engineering Chongqing University of Science and Technology Chongqing China

**Keywords:** hydrogen production, nickel‐based catalysts, reaction mechanisms, tungsten catalyst design, urea oxidation reaction

## Abstract

The urea oxidation reaction (UOR), as a multi‐electron coupling reaction, provides an efficient route for treating urea‐containing wastewater and a sustainable strategy for energy‐saving H2 production. Nickel‐based catalysts attract attention due to their low cost and favorable activity, but single‐metal catalysts still suffer from limited activity and stability. To overcome these limitations, researchers enhance performance through doping, structural regulation, and composite strategies. Tungsten (W), with high electronegativity, multivalence, excellent conductivity, and environmental friendliness, is widely incorporated into nickel‐based catalysts to modulate electronic structure, improve adsorption and conversion of intermediates, and enhance stability. This review summarizes recent progress in W‐based UOR catalysts, focusing on reaction mechanisms, structural regulation strategies, and performance improvement approaches. Current challenges include active site identification, catalyst stability, reaction selectivity, and the lack of standardized evaluation protocols. Future research directions involve elucidating catalytic mechanisms, optimizing material design, establishing unified evaluation standards, and developing high‐performance W‐based composites, aiming to advance UOR applications in energy conversion, wastewater treatment, and sensing.

## Graphical abstract

This work presents tungsten‐modified nickel‐based catalysts for the urea oxidation reaction, highlighting strategies to boost catalytic performance. It focuses on tuning electronic structure, enriching accessible active sites, and designing advanced catalyst structures, providing guidance for the rational development of high‐performance UOR catalysts for energy conversion, wastewater treatment, and sustainable hydrogen production.

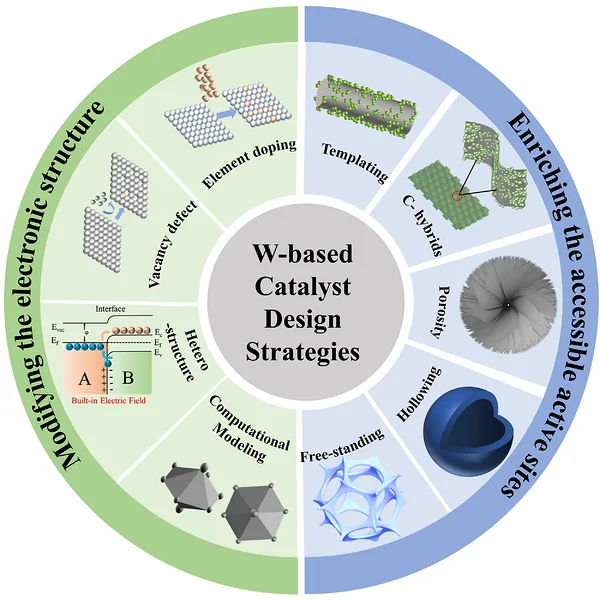

## 1 Introduction

Urea, a ubiquitous nitrogen‐containing chemical, is indispensable in modern industry and agriculture, particularly as a dominant nitrogen‐release fertilizer [1, 2, 3, 4, 5, 6]. However, its extensive application and industrial discharge have led to widespread environmental accumulation. Its subsequent degradation into ammonia and nitrate compounds poses severe risks to ecosystems and human health. Traditional remediation strategies for urea‐rich wastewater, such as pyrolytic hydrolysis, physical adsorption, and chemical oxidation, are constrained by high energy input or stringent operational requirements [7, 8, 9, 10, 11]. Natural urea hydrolysis is kinetically sluggish, and while enzymatic hydrolysis via urease is exceptionally fast, it generates secondary pollutants like NH_4_^+^ and CO_3_^2−^/HCO^3−^, requiring complex downstream processing [12, 13]. Consequently, developing efficient, eco‐friendly, and sustainable urea degradation technologies is imperative. The electrocatalytic urea oxidation reaction (UOR) has emerged as a highly promising alternative [14, 15, 16, 17, 18, 19, 20]. UOR converts urea into benign products under relatively mild conditions, offering a green route for wastewater remediation. Concurrently, urea's high hydrogen content, exceptional water solubility, high volumetric energy density (16.9 MJ L^−1^), and non‐toxic, non‐flammable nature makes it an ideal hydrogen carrier for sustainable energy systems [21].

In the conventional alkaline water electrolysis, hydrogen is generated with the oxygen evolution reaction (OER) at anode Equations (1) and (2) [22, 23, 24, 25]:

(1) $$4OH^{-}\to O_{2}+2H_{2}O+4e^{-}$$

(2) $$2H_{2}O+2e^{-}\to H_{2}+2OH^{-}$$

While the theoretical decomposition voltage is 1.23 V, practical systems operate at ∼1.8 V due to severe anodic polarization and the sluggish multi‐electron kinetics of the OER [26, 27, 28]. Replacing the thermodynamically demanding OER with the oxidation of more readily oxidizable urea molecules significantly lowers the energetic barrier Equations (3) and (4) [29, 30, 31]:

(3)

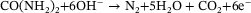

(4) $$6H_{2}O+6e^{-}\to 3H_{2}+6OH^{-}$$

Here, CO_2_ is utilized to represent the carbon‐containing oxidation product in the overall electrochemical reaction of UOR, which is consistent with conventional UOR descriptions. Under strongly alkaline conditions, however, the generated CO_2_ can rapidly react with OH^−^ to form CO_3_^2−^, which is the predominant dissolved carbon species in the electrolyte. Therefore, CO_2_ and CO_3_^2−^ are used to distinguish the carbon‐containing product in the electrochemical reaction pathway and the predominant dissolved carbon species in alkaline media, respectively.

Under standard conditions, the theoretical voltage for urea electrolysis is merely 0.37 V [32, 33, 34, 35, 36]. Thus, replacing OER with UOR holds profound dual significance: it simultaneously remediates urea‐rich wastewater and substantially mitigates the energy consumption of electrolytic hydrogen production. Nevertheless, UOR involves a complex six‐electron transfer process characterized by intrinsic kinetic sluggishness, necessitating highly efficient and robust electrocatalysts to lower the reaction energy barrier [37, 38, 39].

Early UOR research predominantly relied on noble metals which, despite their high activity, are fundamentally limited by elemental scarcity and prohibitive costs [40, 41, 42, 43, 44]. Consequently, the focus has shifted toward cost‐effective, non‐noble transition metal electrocatalysts. Since the seminal discovery in 2009 that nickel (Ni)‐based catalysts effectively drive UOR alongside H_2_ production, Ni has garnered widespread attention [45]. Under alkaline conditions, the dynamically generated NiOOH species has been confirmed as the primary active phase for UOR [46, 47]. However, monometallic Ni catalysts suffer from intrinsic limitations, including a restricted density of exposed active sites, suboptimal electrical conductivity, and susceptibility to structural degradation during polarization. To overcome these bottlenecks, researchers have extensively explored catalysts design strategies such as elemental doping, defect engineering, and heterostructure construction [48, 49, 50, 51, 52, 53, 54].

Among heteroatom modulators, tungsten (W) has emerged as a particularly compelling dopant, characterized by high electronegativity, multivalent electronic configuration, excellent intrinsic conductivity, and environmental benignity [55, 56]. Incorporating W into Ni‐based frameworks effectively tunes the electronic structure of adjacent metal centers, optimizing the electron density at active sites. This modulation can regulate the interaction between active sites and reaction intermediates, thereby mitigating intermediate poisoning and accelerating reaction kinetics [57, 58]. For example, Zeng et al. [59] constructed binder‐free Ni‐W electrodes through a potentiostatic electrodeposition strategy and systematically investigated the role of W incorporation in regulating the electronic structure and UOR performance of Ni‐based catalysts. Experimental and theoretical analyses demonstrated that W incorporation induced electronic redistribution around Ni active sites, enhanced the metallic character of the catalyst, and optimized Ni‐O orbital hybridization. Furthermore, DFT calculations revealed that W incorporation modulated the local electronic structure of Ni active centers and altered the interaction between catalyst surfaces and key reaction intermediates during UOR, thereby promoting the UOR process. This study highlights the critical role of W as an electronic modulator in tailoring the local chemical environment of Ni‐based active sites and improving UOR catalytic performance. Furthermore, W‐based species and metal tungstates provide robust structural stability, tunable micro‐morphologies, and enhanced durability under harsh alkaline oxidative conditions. Consequently, W‐integrated Ni‐based catalysts have recently emerged in the rational design of high‐performance, sustainable UOR catalytic systems [60, 61, 62, 63, 64].

This review systematically summarizes the recent progress in W‐based catalysts for UOR. We first discuss the fundamental mechanisms of UOR and the unique role of W in modulating the electronic and structural properties of Ni‐based active sites. Subsequently, we critically evaluate state‐of‐the‐art catalysts design strategies aimed at boosting performance, including heteroatom doping, defect engineering, heterostructure construction, carbon‐based hybridization, and the development of hollow or self‐supporting structures. Furthermore, we analyze prevailing challenges in the field, such as doubts in active site identification, long‐term stability under operating conditions, reaction selectivity, and the lack of standardized evaluation protocols. Finally, we provide prospective directions aimed at fundamental mechanistic understanding and the practical deployment of high‐performance W‐based catalysts for sustainable energy conversion and environmental remediation.

## 2 Mechanism of UOR

Electrochemical urea oxidation is a multi‐step, proton‐coupled electron transfer process whose pathway is deeply influenced by electrolyte composition, pH, applied potential, and the architecture of the catalytic active sites [65, 66, 67]. These environmental variables dictate the adsorption configuration of urea molecules, shape the path of intermediate formation, and ultimately determine the distribution of final products. In acidic and neutral electrolytes, UOR kinetics are typically sluggish. A simplified overall reaction representation for acidic UOR is given as follows:

(5) $$\mathrm{CO}{\left(NH_{2}\right)}_{2}+H_{2}O\to N_{2}+CO_{2}+6H^{+}+6e^{-}$$

Acidic conditions suffer from insufficient OH^−^ availability, which limits the OH^−^‐assisted dehydrogenation of urea molecules and C‐N bond cleavage processes that are favored in alkaline media [14, 68, 69]. Moreover, many metal oxide catalysts suffer from severe instability in acidic media, undergoing dissolution or reduction that strips them of their ability to activate urea. As summarized by Wang et al. neutral systems similarly suffer from limited OH^−^ availability, leading to sluggish proton‐coupled electron transfer kinetics and consequently impeding the overall reaction process [70]. Halide anions, such as Cl^−^, present in some neutral electrolytes can also competitively bind to active sites or induce electrode corrosion [71, 72, 73]. Conversely, the abundant OH^−^ concentration in alkaline electrolytes serves as a crucial reactant, actively promoting urea dehydrogenation and C‐N cleavage. This markedly accelerates the reaction rate and steers product selectivity. Due to these kinetic and thermodynamic advantages, alkaline electrocatalytically UOR has become the focal point of current research, with most mechanistic investigations anchored in this regime.

The classic UOR mechanism (Pathway 1, Scheme 1), demonstrated by Botte et al. [47], delineates a sequence of adsorption, dehydrogenation, and bond cleavage events. The process initiates with the urea molecule adsorbing onto Ni active sites in a bridging configuration. The carbonyl oxygen and amino nitrogen atoms coordinate to adjacent Ni sites, effectively activating the molecule. The adsorbed urea subsequently undergoes an initial dehydrogenation step Equations (6) and (7), followed by further stepwise dehydrogenation assisted by local OH^−^ groups to form a key *CON_2_ intermediate Equation (8) and (9).

(6)

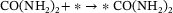



(7)

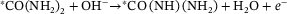

(8) $$*\mathrm{CO}(\mathrm{NH})(NH_{2})+OH^{-}\to *\mathrm{CO}(\mathrm{NH})(\mathrm{NH})+H_{2}O+e^{-}$$

(9) $$*\mathrm{CO}(\mathrm{NH})(\mathrm{NH})+OH^{-}\to *\mathrm{CO}N_{2}+H_{2}O+e^{-}$$

**SCHEME 1:**
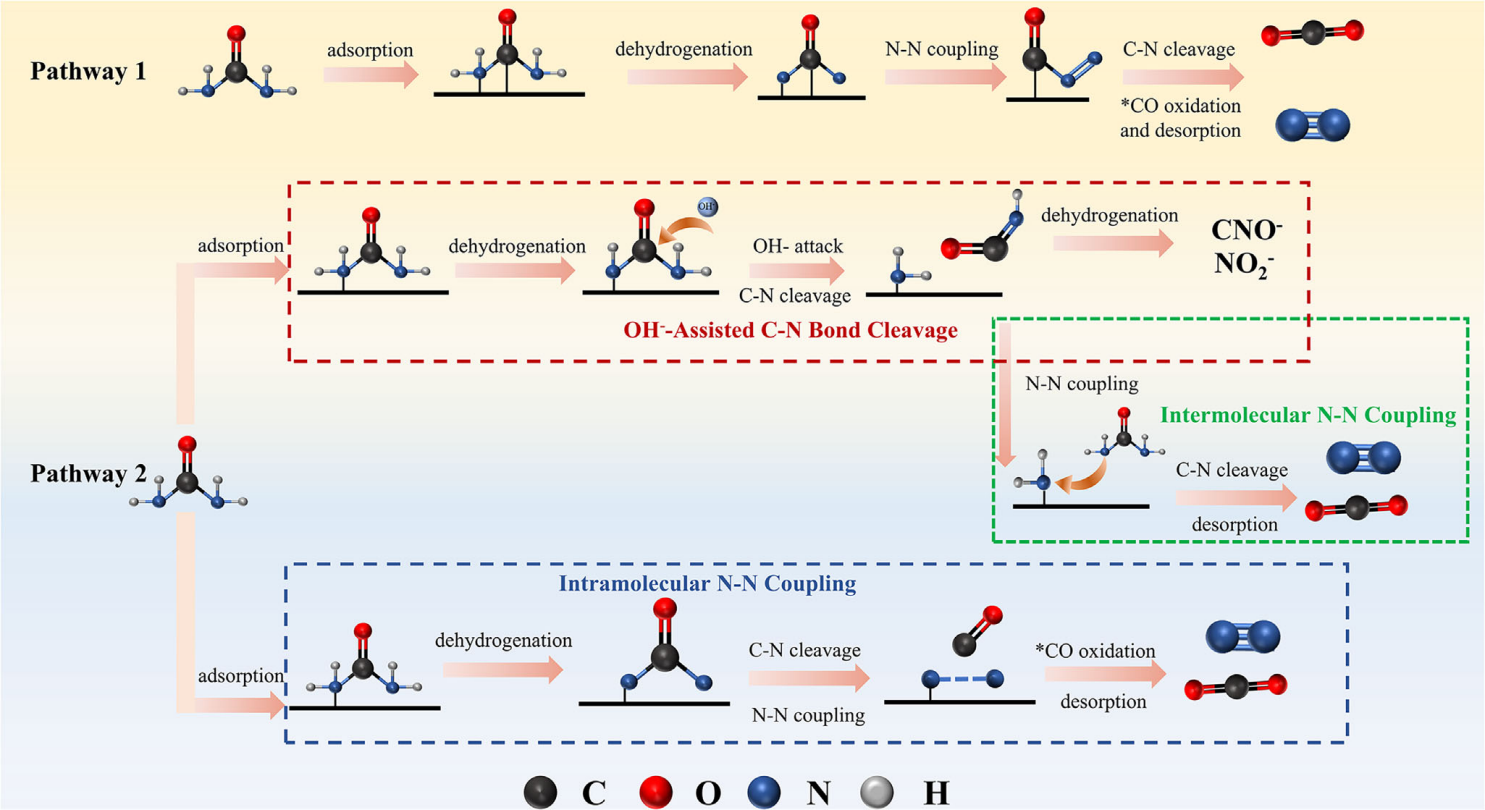
Schematic of possible reaction pathways for the alkaline UOR.

To reach the *CON_2_ state, the molecule undergoes C‐N bond cleavage and simultaneous N‐N coupling, releasing nitrogen gas (N_2_) while leaving a *CO intermediate bound to the catalyst surface Equation (10). This adsorbed *CO species is further oxidized to CO_2_ with the participation of surface hydroxyl groups, ultimately desorbing from the active site Equation (11). Theoretical models suggest that CO_2_ desorption is among the most energetically demanding steps and may represent the rate‐determining step for this pathway. The culmination of these individual steps represents the idealized overall UOR under alkaline conditions Equation (11).

(10) $$*\mathrm{CO}N_{2}\to N_{2}+*\mathrm{CO}$$

(11) $$*\mathrm{CO}+2OH^{-}\to CO_{2}+H_{2}O+2e^{-}+*$$

(12)

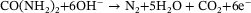




Recent advancements have revealed an alternative mechanistic landscape (Pathway 2, Scheme 1), primarily conceptualized by Li et al. and Tatarchuk et al. [67, 74], which diverges based on the specific interaction of intermediates. This pathway begins with urea adsorption onto the oxidized NiOOH surface, followed by dehydrogenation at a nitrogen atom to yield an *NHCONH_2_ intermediate. From this juncture, the reaction can cascade down three distinct branching routes depending on the local kinetic environment. The first and most kinetically dominant route is the OH^−^‐assisted C‐N bond cleavage. Here, OH^−^ executes a nucleophilic attack on the carbon center of the urea molecule, forming an HNCO(NH_2_)OH intermediate. After an exothermic conformational shift, the C‐NH_2_ bond cleaves, generating *NH_2_ and *COONH. The *NH_2_ species proceeds to oxidize into NO_2_^−^ and partially NO_3_^−^, while *COONH dehydrogenates to produce CNO. This route exhibits the lowest energy barrier in alkaline systems, meaning it can achieve high Faradaic efficiencies for NO_2_^−^, with carbonate species representing the corresponding carbon‐containing product under alkaline conditions Equation (13). However, this high efficiency comes at the cost of overoxidation, generating byproducts that pose secondary environmental risks.

(13)

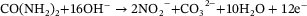




The second and third branching routes of Pathway 2 involve nitrogen coupling, either intra‐ or intermolecularly. In the intramolecular N─N coupling route, the *NHCONH_2_ intermediate undergoes further nitrogen dehydrogenation to form NCONH. A subsequent proton‐coupled electron transfer transforms this into a cyclic *NNH‐CO species, which is finally oxidized to produce N_2_ and carbonate species under alkaline conditions Equation (14). While this route boasts excellent product selectivity towards benign N_2_, its high energy barrier intrinsically limits its overall kinetic contribution. Alternatively, the intermolecular N‐N coupling route occurs when two adjacent *NHCONH_2_ molecules couple via their ‐NHx groups. This interaction generates cross‐products (e.g., ^14^N^15^N or 1^14^N^15^NO) that eventually desorb as N_2_ or N_2_O. This bimolecular route successfully enhances N_2_ selectivity and suppresses NO_2_^−^ formation. Furthermore, if NO_2_^−^ intermediates are already present, a reaction akin to selective catalytic reduction may ensue, yielding N_2_O. Though intermolecular coupling plays a relatively minor role in the total reaction flux, it highlights the complex, potentially recursive nature of UOR intermediates.

(14) $$\mathrm{CO}{\left(NH_{2}\right)}_{2}+8OH^{-}\to N_{2}+{\mathrm{CO}}_{3}^{2-}+6H_{2}O+6e^{-}$$

Therefore, the UOR is an intricate nexus of multi‐electron transfers, selective C─N bond cleavages, and dynamic intermediate formations. The dominant trajectory is highly sensitive to the electrolyte pH and the precise electronic and geometric structure of catalytic active sites. A nuanced understanding of these competing pathways is indispensable for rationally designing catalysts that optimize reaction efficiency and steer product selectivity towards harmless effluents. In this pursuit, incorporating W, with its high electronegativity, exceptional conductivity, and variable valency, into nickel or other transition‐metal matrices offers a powerful lever. W doping dynamically modulates the catalyst's electronic structure, optimizing the binding energy of challenging intermediates like *CO and *NH_2_, thereby facilitating their conversion while stabilizing the high‐valence active phases required to drive complex pathways efficiently.

## 3 Strategies for Boosting UOR Performance

### 3.1 Modifying the Electronic Structure

Catalytic activity is fundamentally determined by the interaction strength between metal active sites and reaction intermediates during the electrocatalytic UOR. If the intermediates bind too strongly to the active sites, desorption tends to be hindered and site blocking may occur; conversely, excessively weak binding impedes efficient adsorption and activation of urea molecules. Hence, maintaining a favorable balance between adsorption and desorption of key intermediates while ensuring sufficient activation of reactants is a critical factor for enhancing the intrinsic catalytic activity of UOR. Modulating the electronic structure of catalysts is widely recognized as an effective strategy to improve intrinsic activity [75]. By altering the electron density, valence distribution, and d‐orbital energy levels of metal centers, the adsorption strength for reaction intermediates and the reaction energy barriers can be significantly influenced, thereby regulating the reaction pathway and rate‐determining steps. Accordingly, researchers have systematically tuned the electronic structure of Ni‐based catalysts through strategies such as heteroatom doping, defect engineering, and heterostructure construction (Scheme 2). With the assistance of theoretical calculations, the effects of electronic state distribution, adsorption behavior, and reaction energy barriers have been further illuminated, thus providing guidance for the rational design of efficient UOR catalysts.

**SCHEME 2:**
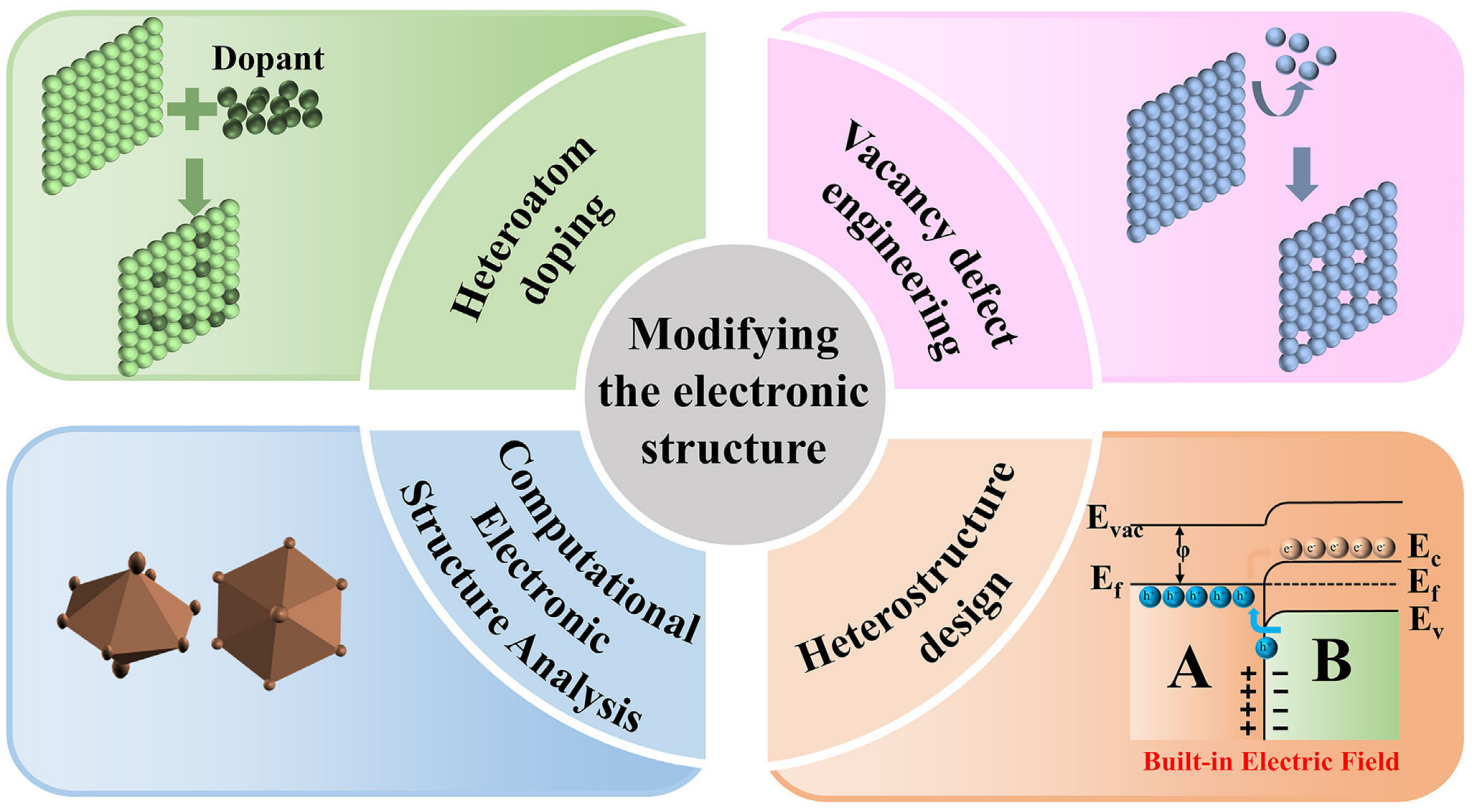
Schematic illustration of electronic regulation strategies for W‐based UOR electrocatalysts.

#### 3.1.1 Heteroatom Doping

Heteroatom doping is a highly efficient strategy to induce targeted electronic reconstruction in Ni‐based catalysts. By introducing elements with distinct electronegativities and atomic radii, the local coordination environment and d‐band center of the active metal can be precisely tuned. Tungsten (W), with its strong electron‐withdrawing nature and multivalent states, is particularly adept at modifying the Ni─O bond strength and modulating the binding energies of reaction intermediates, effectively avoiding the repetitive structural challenges seen in single‐metal systems. For instance, Liao et al. [76] designed a hybrid catalyst by growing NiFe‐layered double hydroxide (LDH) nanosheets on oxygen‐deficient WO_3‐x_ nanorods (NiFe LDH/WO_3‐x_) (Figure 1a,b). This architecture delivered a current density of 20 mA cm^−2^ at a low potential of 1.43 V (Figure 1c) and exhibited stable operation over 50 h. The performance enhancement is fundamentally driven by the electronic synergy between the support and the LDH; XPS and in situ Raman spectroscopy (Figure 1d) revealed that electron transfer from Ni/Fe to the W sites significantly increases the Ni^3+^/Ni^2+^ ratio, accelerating the formation of the crucial NiOOH active phase. Furthermore, the W─O sites mitigate intermediate poisoning, thereby extending the operational lifespan of the electrocatalyst.

**FIGURE 1:**
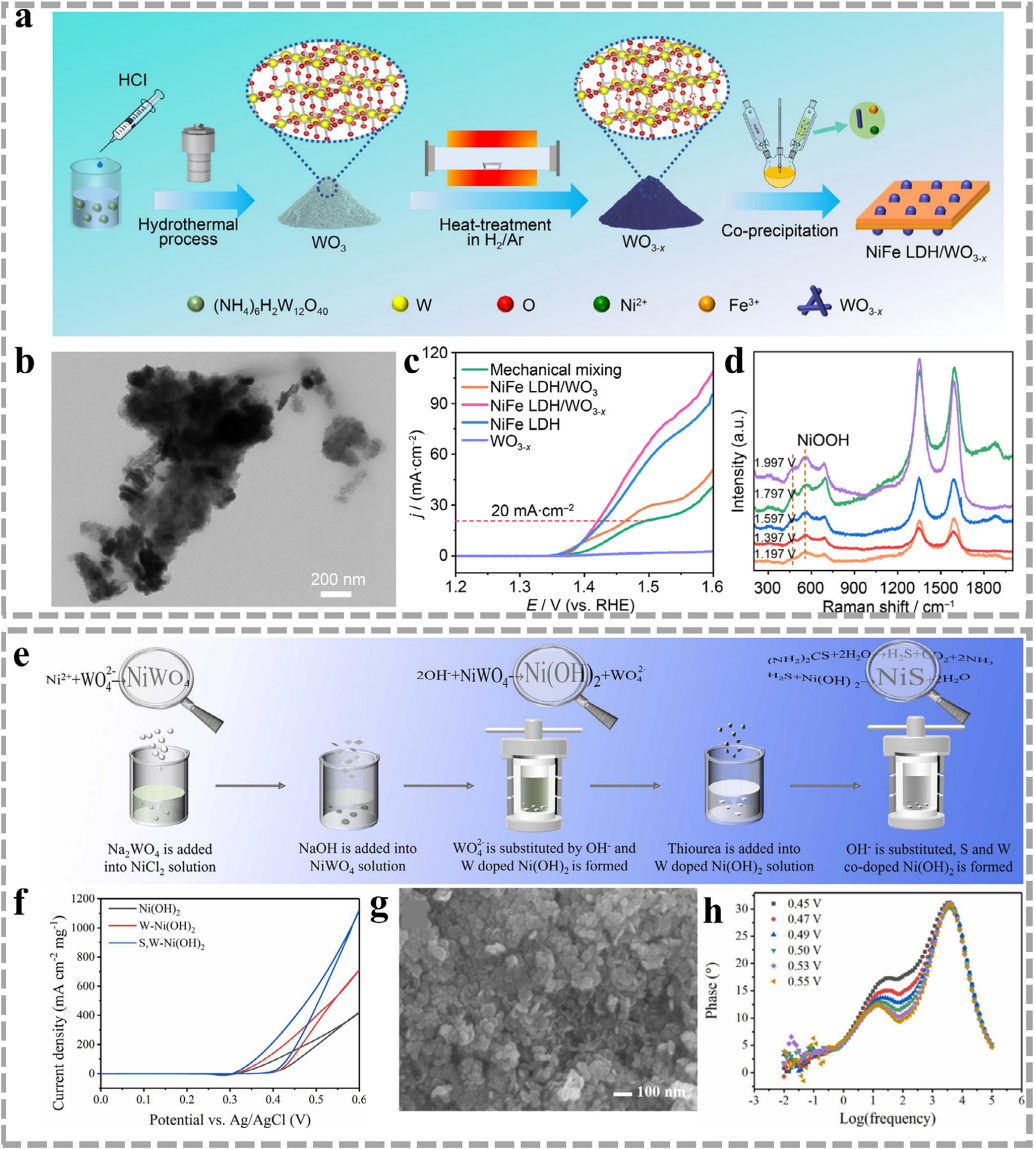
(a) Preparation illustration of NiFe LDH/WO_3‐x_, (b) TEM image of NiFe LDH/WO_3‐x_, (c) LSV curves of different samples in 1 M KOH and 0.33 M urea, (d) Raman spectra tested at different working potential. Reproduced with permission [76]. Copyright 2024, Springer Nature. (e) The preparation illustration of S, W‐Ni(OH)_2_, (f) CV curves of different samples in 1 M KOH and 0.33 M urea, (g) SEM image of S, W‐Ni(OH)_2_, (h) Nyquist plots of S, W‐Ni(OH)_2_ at various potentials. Reproduced with permission [77]. Copyright 2023, Elsevier.

Building on single‐element modulation, synergistic co‐doping offers more sophisticated control over the local electronic environment. Yang et al. [77] demonstrated this through sulfur and tungsten co‐doped Ni(OH)_2_ nanosheets (S,W‐Ni(OH)_2_, Figure 1e). Crucially, direct synthesis of pure NiWO_4_ yielded negligible UOR activity, proving that W must be incorporated directly into the Ni(OH)_2_ lattice to function as an effective electronic dopant. The S, W‐Ni(OH)_2_ catalyst delivered enhanced UOR activity and reduced Tafel slopes compared to single‐doped or pristine counterparts (Figurs 1f,g). Notably, electrochemical impedance spectroscopy (EIS) indicated that W doping continuously decreases charge‐transfer and adsorption‐related resistances with increasing potential. This behavior is associated with the improved resistance toward intermediate poisoning, as W species weaken the excessive adsorption of CO* intermediates on Ni^3+^ active sites, thereby maintaining accessible catalytic sites during UOR (Figure 1h).

While these studies highlight the efficacy of W doping in promoting the Ni^2+^ to Ni^3+^ transition and mitigating intermediate poisoning, several underlying mechanisms require deeper scrutiny. First, the exact role of W, whether it strictly acts as a distal electronic modulator for Ni sites or dynamically participates in the intermediate binding itself, remains ambiguous in multi‐component systems. Additionally, while the W‐induced electronic deficit on Ni clearly lowers the energy barrier for the formation of NiOOH, the long‐term thermodynamic stability of high‐valence W dopants under extreme alkaline anodic conditions is rarely addressed. Prolonged anodic polarization often induces leaching of highly soluble transition metal oxo‐anions. It is valuable to track the dynamic dissolution or phase segregation of W species during prolonged electrolysis. This is critical to ensure that initial activity enhancements derived from doping do not mask long‐term structural degradation.

#### 3.1.2 Vacancy Defect Engineering

Vacancy defect engineering systematically disrupts local crystallographic symmetry, moving beyond straightforward elemental substitution. Deliberately engineering structural voids, either as anion or cation vacancies, generates highly localized, electron‐rich or electron‐deficient microenvironments that profoundly restructure the energetic landscape for adsorbate interactions, thereby accelerating multi‐electron UOR kinetics [78].

In anion vacancy engineering, particularly concerning oxygen vacancies, the incorporation of high‐valence dopants into a host lattice naturally forces lattice distortion and the robust generation of oxygen vacancies to maintain charge neutrality. This phenomenon fundamentally shifts the host's electronic signature. For example, forming W‐doped Ni(OH)_2_/NiO core‐shell arrays induces significant defect sites that act as electron‐donating centers (Figure 2a) [79]. W incorporation coupled with phosphate modification regulates the localized electronic environment of adjacent Ni sites, enhancing electron delocalization and improving the metallic character of the catalyst. Such electronic modulation facilitates charge transfer, promotes urea adsorption and subsequent activation, and lowers the energy barriers associated with PCET processes and C‐N bond cleavage. (Figure 2b,c). Furthermore, the coupling of W^6+^ integration and abundant oxygen vacancies acts synergistically to stabilize the high‐valence NiOOH active phase, preventing over‐oxidation and structural collapse while accelerating the rate‐determining C─N bond cleavage step (Figure 2d). The resulting self‐supported, defect‐rich arrays consistently maintain low overpotentials and improved long‐term stability, with reduced susceptibility to intermediate poisoning.

**FIGURE 2:**
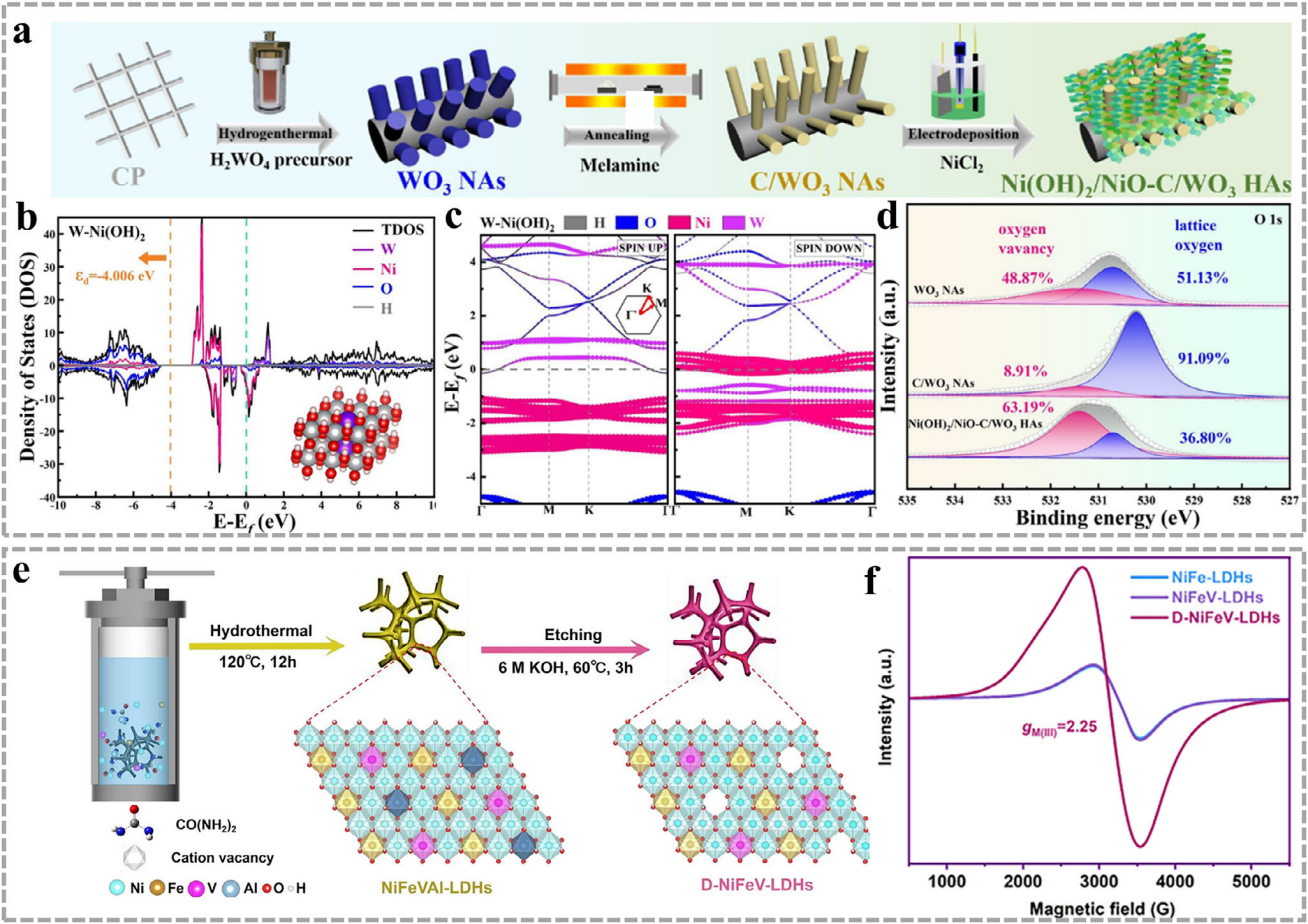
(a) Scheme of the synthesis of the Ni(OH)_2_/NiO‐C/WO_3_ HAs, (b) calculated total and partial density of states for W‐Ni(OH)_2_, (c) calculated band structures of W‐Ni(OH)_2_, (d) high‐resolution XPS spectra of O 1 s for different samples. Reproduced with permission [79]. Copyright 2024, Elsevier. (e) Scheme of the preparation procedure for D‐NiFeV‐LDHs supported on nickel iron foam, (f) EPR spectra (c) of NiFe‐LDHs. Reproduced with permission [80]. Copyright 2024, Elsevier.

Conversely, cation vacancy engineering focuses on the strategic removal of metal nodes within the lattice framework. This can be effectively achieved by coupling multivalent heteroatom doping with selective chemical etching. When alkali‐soluble metal species within LDHs are leached out, the resulting metal cation vacancies create highly unsaturated coordination environments (Figure 2e) [80]. The physical absence of metal cations forces an intense electronic rearrangement of the surrounding lattice oxygen, easily observable through distinct paramagnetic resonance signals indicative of unpaired electrons (Figure 2f). When combined with adjacent V‐doping, these geometric defects trigger a massive redistribution of surface charge density, forcing the Ni 3d‐band center upward toward the Fermi level. This deliberate disruption enhances the electronic interaction between active sites and adsorbed urea species, thereby facilitating initial urea activation and improving charge‐transfer characteristics.

Beyond heteroatom doping‐induced oxygen vacancy formation, controlled thermal reduction provides another effective strategy for constructing defect‐rich tungsten‐based catalysts. For example, partial thermal reduction of NiCoWO_x_ electrodes selectively removes lattice oxygen to generate abundant oxygen vacancies (Figure 3a) [81]. These defects regulate the local electronic environment, improve charge transfer properties, and facilitate the formation of high‐valence Ni/Co active species, thereby modulating urea adsorption and activation and accelerating UOR kinetics (Figure 3b). Furthermore, amorphous structures offer a new approach for stabilizing and dynamically regulating oxygen vacancies. Oxygen vacancy‐rich amorphous NiWOx can accommodate and maintain a high concentration of defects through its disordered atomic arrangement, while enabling continuous vacancy generation and regeneration during the reaction process (Figure 3c) [82]. The enriched oxygen vacancies promote the formation of active NiOOH species and regulate the electronic structure of Ni centers, enhancing urea activation (Figure 3d). Theoretical calculations further reveal that oxygen vacancy regulation lowers the energy barrier of the rate‐determining C‐N bond cleavage step, thereby improving reaction kinetics (Figure 3e,f). This work highlights the synergistic effect between amorphization and defect engineering in achieving efficient and durable urea oxidation while maintaining structural defect stability.

**FIGURE 3:**
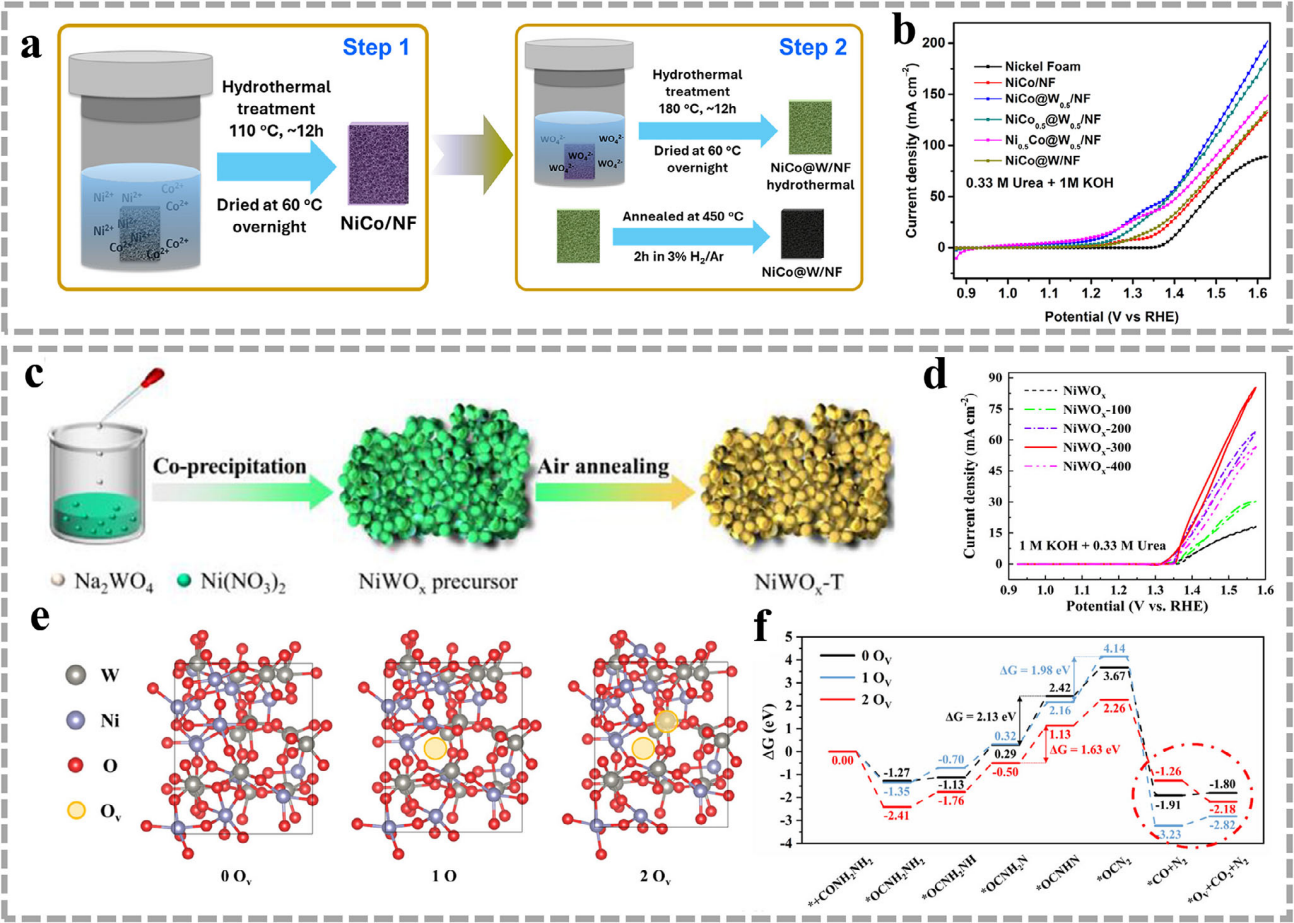
(a) Schematic of synthesis procedure of NiCoWO_x_ materials on Ni Foam, (b) LSV curves of different catalysts for UOR. Reproduced with permission [81], published by MDPI. Copyright 2026 by the authors. (c) Schematic diagram of the synthesis of NiWO_x_‐T catalysts, (d) CV curves of the NiWO_x_ precursor and amorphous NiWO_x_‐T, (e) the geometric structure of the amorphous NiWO_x_ with and without O_v_, (f) Gibbs free energy diagrams for the UOR on the amorphous NiWO_x_ with and without O_v_. Reproduced with permission [82]. Copyright 2026, Wiley.

While the literature overwhelmingly reports that manipulating vacancy defects enhances intrinsic UOR activity, several critical aspects require deeper mechanistic scrutiny. First, because defect sites are intrinsically thermodynamically unstable, high‐energy states, their dynamic, *operando* evolution under harsh, highly oxidative alkaline conditions remains a major blind spot; it is unclear whether these engineered oxygen or cation vacancies persist during prolonged UOR electrolysis or undergo progressive passivation via irreversible filling by electrolyte hydroxide ions, necessitating advanced operando characterizations like dynamic XAFS to track defect lifetimes. Furthermore, because both anion and cation defect strategies often couple vacancy generation with the introduction of heteroatoms, current trends frequently conflate the isolated electronic contribution of the dopant with the structural impact of the vacancy. Consequently, advanced theoretical frameworks and microkinetic modeling must be developed to decouple these variables and establish precise structure‐activity relationships. Finally, the prevailing “more is better” pattern regarding defect concentration must be re‐evaluated, as excessive vacancy engineering inevitably degrades lattice integrity and creates charge‐carrier recombination centers that paralyze bulk electrical conductivity.

#### 3.1.3 Heterostructure Design

Beyond singular phase modulation, constructing engineered heterointerfaces, particularly Schottky‐type junctions, introduces a powerful macroscopic driving force for electronic reorganization. When two distinct phases are coupled, the differential in their work functions inherently generates a built‐in electric field. This field mandates spontaneous, directional charge transfer across the interface until Fermi level equilibration is achieved. In the context of UOR, this interfacial polarization physically dictates the electron density of the catalytic centers, thereby fine‐tuning the chemisorption of nucleophilic functional groups and substantially fortifying the mechanical and physicochemical resilience of the hybrid matrix [83].

The deliberate coupling of W and Ni species at the nanoscale enables radical mechanistic shifts [84]. For example, integrating WN with Ni_3_C creates an interface where charge dynamically funnels from the WN phase to the Ni_3_C domain (Figure 4a). This specific charge accumulation stabilizes the local Ni sites in lower valency states (around +2, Figure 4b). Rather than relying on the traditional generation of high‐valence NiOOH, this unique electronic environment fundamentally alters the UOR pathway. Theoretical frameworks confirm that the W sites at this heterointerface dramatically lower the activation energy required to transition from the *CO‐N_2_ intermediate to *CO·OH (Figure 4c). Consequently, the reaction bypasses the energetically demanding NiOOH‐mediated route, unlocking a highly efficient alternative pathway that sustains high‐current density operation with minimal degradation.

**FIGURE 4:**
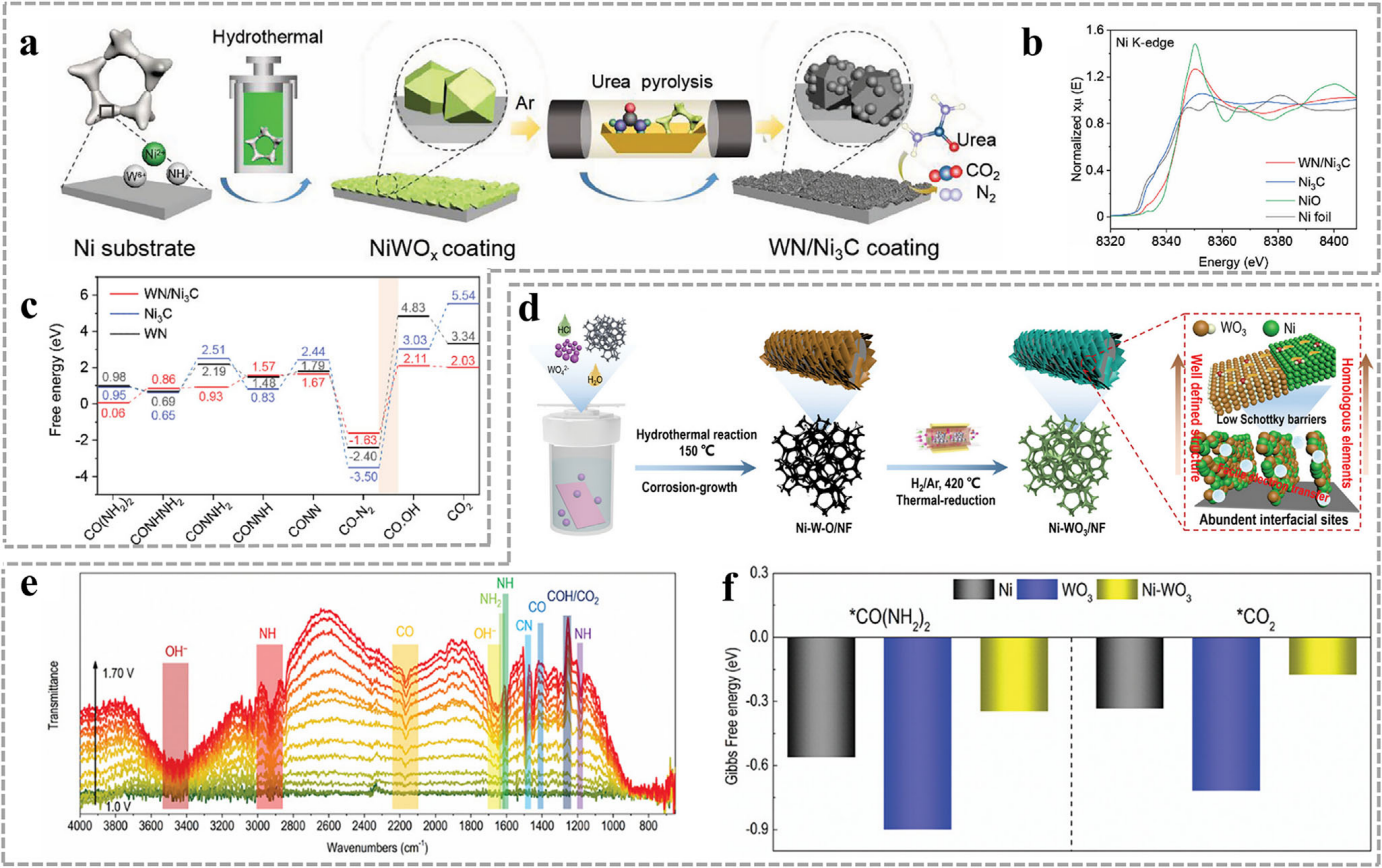
(a) Synthetic scheme of an active WN/Ni_3_C ceramic coating electrocatalyst, (b) XANES spectra at Ni K‐edge for different samples, (c) the free energy diagram of UOR from urea molecule to N_2_ and CO_2_ at the W site of different samples. Reproduced with permission [84]. Copyright 2023, Wiley. (d) Schematic illustration of the formation process of Ni‐WO_3_ Schottky heterojunction on nickel foam, (e) in situ FTIR spectra of Ni‐WO_3_/NF electrode of UOR, (f) free energy diagram for adsorption of urea molecule and CO_2_ molecule during the UOR process. Reproduced with permission [85]. Copyright 2024, Wiley.

Conversely, engineering a heterointerface where the tungsten phase possesses a significantly higher work function than the nickel phase, such as in a Ni‐WO_3_ hybrid, reverses the directional charge flow (Figure 4d) [85]. The resulting Schottky junction forcefully extracts electrons from the adjacent Ni atoms, rendering them acutely electron‐deficient. This engineered electron depletion acts as a thermodynamic springboard, vastly accelerating the anodic oxidation of Ni to the catalytically indispensable NiOOH phase. Furthermore, the electronic interplay across the Ni‐WO_3_ boundary regulates the adsorption/desorption behavior of key UOR intermediates, resulting in balanced interactions between active sites and reaction species. The adsorption strength of urea molecules is regulated to provide sufficient interaction for activation while avoiding excessive binding, whereas the weakened interaction with the *COO intermediate facilitates its subsequent desorption. This balanced modulation of intermediate binding promotes favorable reaction kinetics and accelerates the overall UOR process (Figure 4e,f).

The introduction of carbide phases provides an effective approach to regulate transition‐metal active centers through interfacial electronic coupling. For example, the WC@Co(OH)_2_ heterostructure induces interfacial charge redistribution, promotes the formation of high‐spin Co^3+^ species, and modulates the electronic structure of Co sites, thereby enhancing UOR kinetics (Figure 5a,b) [86]. Similarly, the Ni_2_P/WP/W ternary heterostructure regulates the electronic environment of Ni centers through interfacial coupling between W‐based components and Ni_2_P (Figure 5c) [87]. Theoretical calculations further reveal that W‐modulated active surfaces regulate urea adsorption characteristics and reduce the energy barrier for C─N bond activation (Figure 5d). Furthermore, strong metal‐support interaction (SMSI) offers an additional pathway for interfacial electronic regulation. For instance, the NiCo‐Na_0.3_WO_3_heterostructure adjusts the electronic states of NiCo active sites through electron coupling between the support and alloy phases (Figure 5e,f) [88]. This interfacial reconstruction regulates the interaction between active sites and key carbon‐containing intermediates. Theoretical analysis further demonstrates that SMSI overly strong adsorption of CO_2_‐derived species and lowers the desorption energy barrier, thereby mitigating intermediate accumulation and improving UOR stability (Figure 5g).

**FIGURE 5:**
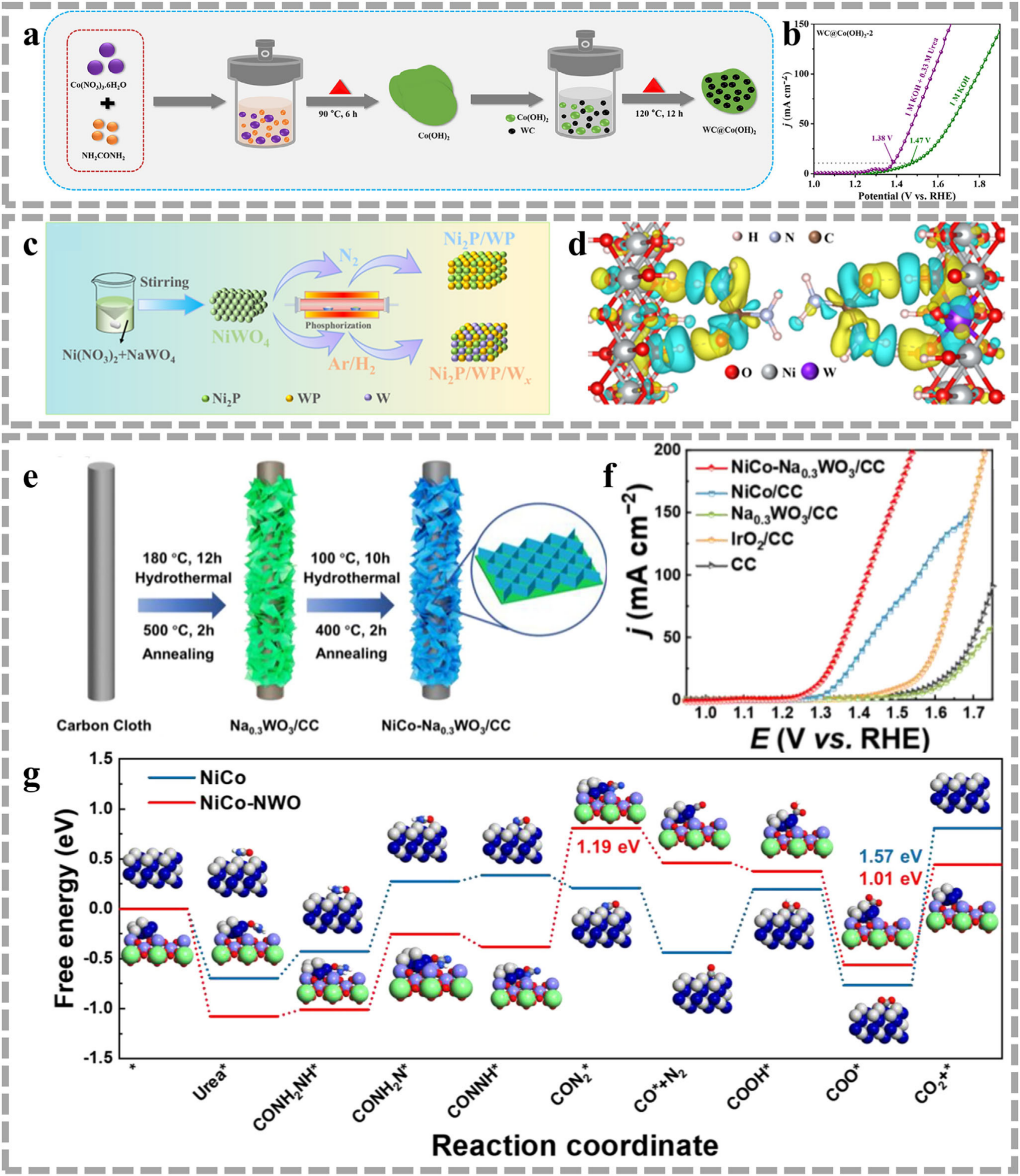
(a) Preparation process of WC@Co(OH)_2_, (b) comparison of OER and UOR performance of WC@Co(OH)_2_‐2. Reprinted with permission [86]. Copyright 2025, Wiley. (c) Schematic illustration of the fabrication of Ni_2_P/WP and Ni_2_P/WP/W_x_, (d) charge density difference of urea adsorbed on (left side) NiOOH and (right side) W‐doped NiOOH. Reproduced with permission [87]. Copyright 2026, Elsevier. (e) NiCo‐Na_0.3_WO_3_/CC synthesis diagram, (f) polarization curves of NiCo‐Na_0.3_WO_3_/CC and its contrast samples, (g) Gibbs free energy of UOR pathway. Reproduced with permission [88]. Copyright 2026, Wiley.

While the literature celebrates the construction of Schottky junctions to manipulate localized electron densities, several inherent limitations must be addressed in future studies. First, the built‐in electric field is fundamentally a thermodynamic construct established at zero bias. Under the intense anodic polarization required for UOR, the dynamic stability of this junction remains largely unexplored. High operating potentials can induce band unpinning, severe surface reconstruction, or phase segregation, which may ultimately collapse the engineered electric field entirely. Second, the spatial extent of the hetero‐effect is strictly confined to a nanometer‐scale interfacial perimeter; the electronic modulation decays exponentially away from the boundary. Maximizing the absolute number of these active boundary sites without causing the distinct nanoscale phases to fuse into a monolithic solid solution remains a formidable synthetic bottleneck. Lastly, while it is theoretically convenient to attribute reactant activation to one phase and intermediate desorption to the other, definitively mapping where the exact catalytic turnover occurs, or whether intermediates physically spill over between the phases, requires sophisticated operando spectroscopic techniques that have yet to be widely applied to W‐based heterostructures.

#### 3.1.4 Computational Insights Into Electronic Structure Modulation

According to the d‐band theory, modulation of the d‐band center regulates the interaction between metal active sites and adsorbed intermediates by altering the orbital overlap between catalyst surfaces and reaction species. Generally, an upward shift of the d‐band center toward the Fermi level strengthens adsorbate binding, whereas a downward shift weakens such interactions. However, the catalytic consequence of electronic structure modulation, including d‐band center shifts, is highly dependent on the specific intermediates and elementary steps involved in the reaction. For UOR, enhanced adsorption can facilitate the activation of reactants or transition states, while excessive binding of strongly adsorbed intermediates may hinder subsequent conversion or desorption. Therefore, either strengthening or weakening metal–adsorbate interactions can contribute to improved catalytic performance when the adsorption strength of rate‐determining intermediates is appropriately regulated. When adsorption energies are discussed, more negative values indicate stronger interactions between adsorbates and catalyst surfaces, whereas less negative values correspond to weaker adsorption and more favorable desorption.

Computational modelling, primarily driven by density functional theory (DFT), transcends empirical observation by providing atomic‐resolution mapping of the UOR energetic landscape. By simulating the adsorption thermodynamics of key intermediates (*OH, *CO, *NH_2_), DFT reveals the structural modifications, such as W‐doping, fundamentally restructure intrinsic reaction coordinates. At the single‐atom limit, introducing isolated W sites into a Ni(OH)_2_ matrix act as an active electron‐regulating hub. Rather than unconditionally driving Ni to the traditional high‐valent NiOOH phase, the localized electrostatic field induced by W dynamically stabilizes a lower‐energy active intermediate, Ni(OH)O [89]. This electronic tuning fundamentally alters the operative mechanism (Figure 6a). By suppressing the thermodynamic feasibility of competing OER, W‐doping simultaneously lowers the activation barriers for critical UOR steps, specifically the nucleophilic attack on the urea molecule and subsequent C─N bond cleavage (Figure 6b). Furthermore, theoretical projections of the d‐band center confirm that synergistic interactions between the W dopant and surrounding anions shift the Ni d‐band closer to the Fermi level (Figure 6c). This upward shift enhances the metallic character of active sites and facilitates interfacial charge transfer. Its catalytic contribution is associated with improved electronic conductivity rather than a universal enhancement of adsorbate binding. Consequently, DFT reveals the rate‐determining step in these engineered systems pivots from complex intermediate dehydrogenation to initial urea adsorption event, accelerating overall reaction kinetics.

**FIGURE 6:**
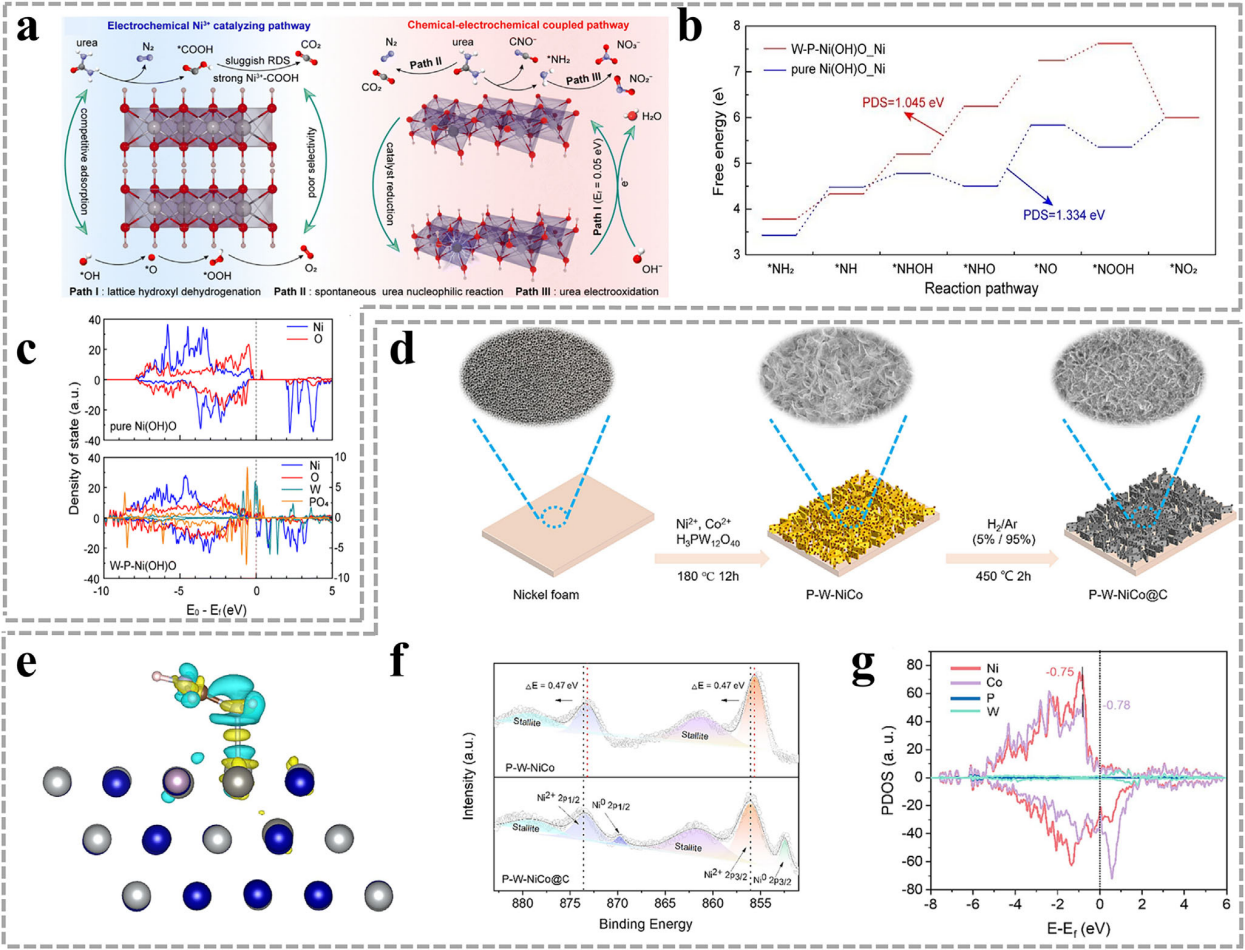
(a,b) Free energy diagram of W‐P‐Ni(OH)O along the E‐E UOR pathway, (c) DOS of pure Ni(OH)O and W‐P‐Ni(OH)O models. Reproduced with permission [89]. Copyright 2025, The Royal Society of Chemistry. (d) Synthetic illustration of P‐W‐NiCo@C, (e) charge density difference of urea adsorbed on P‐W‐ NiCo@C catalysts, (f) XPS spectra of P‐W‐NiCo and P‐W‐NiCo@C, Ni 2p spectra, (g) PDOS of P‐W‐NiCo@C. Reproduced with permission [90]. Copyright 2024, Elsevier.

Beyond isolated dopants, computational models also elucidate the complex electronic interplay within multi‐element matrices, such as P and W co‐doped NiCo alloy frameworks (Figure 6d) [90]. In these heterovalent environments, DFT demonstrates that multi‐element synergistic doping induces profound electronic redistribution (Figure 6e). High‐valent W atoms exert a pronounced electron‐withdrawing effect, actively diminishing the localized electron density around the primary Ni and Co catalytic centers (Figure 6f). This induced electropositivity systematically lowers the formation energy required to generate catalytically active high‐valent phases during anodic polarization. Crucially, partial density of states (PDOS) analyses in these complex alloys reveal that P and W co‐doping induces an upward shift of the Ni/Co d‐band centers, thereby optimizing the electronic structure of active sites (Figure 6g). According to DFT calculations, this electronic modulation enhances charge transfer and strengthens the adsorption of urea molecules at active sites, facilitating urea activation and promoting the UOR process. Consequently, the modified electronic structure contributes to improved UOR kinetics.

Computational modelling has provided profound mechanistic insights; several critical limitations must be addressed to capture the true *operando* complexity of UOR. Primarily, most DFT investigations rely on strict vacuum or simplified implicit solvation models that fail to reflect the dynamically fluctuating hydrogen‐bonding networks of highly concentrated alkaline electrolytes; future efforts must therefore transition toward ab initio molecular dynamics (AIMD) incorporating explicit solvation shells to accurately model the governing proton‐coupled electron transfer (PCET) kinetics. Furthermore, standard static models typically map reaction pathways on idealized, pristine crystallographic facets, largely ignoring the severe electrochemical reconstruction of W‐doped Ni surfaces into amorphous, dynamically shifting hydroxide/oxyhydroxide layers. To accurately simulate these disordered, *operando*‐generated active phases, computational frameworks must evolve beyond static precursor lattices. In this context, emerging machine‐learning‐based methods provide new opportunities to overcome the intrinsic limitations associated with computational scale and structural complexity. Machine‐learned interatomic potentials (MLIPs), trained on high‐quality first‐principles datasets, can approach DFT‐level accuracy while enabling simulations over extended spatial and temporal scales. Such approaches are particularly promising for W‐based UOR catalysts, where they may facilitate the investigation of dynamic surface reconstruction, defect evolution, and interfacial interactions under realistic electrochemical environments. Furthermore, ML‐assisted screening strategies can accelerate the identification of promising W‐based catalyst candidates by establishing correlations between structural descriptors, electronic properties, and catalytic activity, thereby enabling more efficient optimization of dopant configurations and active‐site environments. However, the reliability of these machine‐learning approaches remains strongly dependent on the availability and diversity of training datasets. In particular, the limited representation of amorphous oxyhydroxide phases, dynamically reconstructed surfaces, and charged electrochemical interfaces poses significant challenges for developing accurate predictive models for realistic UOR systems. In parallel, current free energy diagrams overwhelmingly assume low adsorbate coverage limits, which starkly contrasts with functioning electrolyzers where active sites are densely populated by competing *OH, *CO, and unreacted urea molecules. Incorporating coverage‐dependent adsorption energies and lateral adsorbate‐adsorbate interactions into DFT frameworks is thus indispensable for predicting authentic operando catalyst behavior and identifying the precise kinetic onset of intermediate poisoning.

Overall, electronic structure modulation strategies provide diverse approaches for understanding and enhancing the UOR performance of W‐based Ni catalysts. Although these strategies regulate catalytic behavior through different pathways, heteroatom doping primarily modifies the local electronic structure and coordination environment, vacancy engineering alters surface reactivity by introducing unsaturated sites and inducing electronic reconstruction, while heterostructure design further enables interfacial synergistic regulation through electronic coupling between different phases. Meanwhile, computational modelling provides important theoretical support for elucidating the intrinsic relationship between electronic modulation and catalytic kinetics. Collectively, these strategies share a common objective of constructing catalytically favorable electronic environments, thereby regulating the valence evolution of Ni‐based active sites, interfacial charge‐transfer processes, and interactions with key reaction intermediates, which provides fundamental guidance for the rational design of W‐based UOR catalysts (Table 1).

**TABLE 1:** Electronic regulation strategies for W‐based UOR catalysts and their effects on catalytic activity.

Catalysts	Major strategy	Key regulation	Mechanistic role	Refs.
NiFe LDH/WO_3‐x_	W‐induced heteroatom	Charge redistribution; increased Ni^3+^/Ni^2+^ ratio	NiOOH formation; reduced poisoning	[76]
S,W‐Ni(OH)_2_	S/W co‐doping	Weakened CO* adsorption on Ni^3+^ sites	Reduced intermediate poisoning; maintained accessible active sites	[77]
Ni(OH)_2_/NiO C/WO_3_ HAs	Vacancy defect engineering	Oxygen vacancy‐induced electronic modulation	Stabilized active phase and accelerated C‐N cleavage	[79]
D‐NiFeV‐LDHs	Cation vacancy engineering	Up‐shifted Ni 3d‐band center	Enhanced urea activation and charge transfer	[80]
NiCoWO_x_/NF	Oxygen vacancy engineering	Enriched O_v_ and tuned Ni/Co electronic structure	Promoted Ni/Co activation; Accelerated urea conversion	[81]
NiWO_x_‐T	Amorphous structure‐enabled oxygen vacancy engineering	Stabilized Ov with dynamic regeneration	Enhanced NiOOH formation; lowered C‐N cleavage barrier	[82]
WN/Ni_3_C	Heterostructure design	Interfacial charge redistribution	Bypassed NiOOH pathway and lowered *CO‐N_2_ → *CO·OH barrier	[84]
Ni‐WO_3_/NF	Heterostructure design	Interfacial electron depletion	Promoted NiOOH formation and balanced intermediate adsorption	[85]
WC@Co(OH)_2_	Heterostructure engineering	Interfacial charge redistribution	Promotes Co^3+^ active species formation; optimizes UOR kinetics	[86]
Ni_2_P/WP/W	Heterostructure engineering	W/Ni_2_P electronic coupling	Regulates urea adsorption; facilitates C‐N bond activation	[87]
NiCo‐Na_0.3_WO_3_	Heterostructure engineering	SMSI‐induced electron transfer	Weakens excessive CO_2_‐derived species adsorption	[88]

### 3.2 Enriching the Accessible Active Sites

Beyond intrinsic electronic modulation, macroscopic UOR activity is strongly influenced by the volumetric density of electrochemically accessible active sites. For an intricate six‐electron transfer process involving bulky intermediates and multiple gaseous products, catalytic efficiency is inherently bottlenecked by mass transport kinetics. Active centers buried within the bulk lattice or trapped in dense agglomerates remain electrochemically dormant. Consequently, advanced structural engineering, encompassing template‐directed porosification, hollow structures, carbon nano‐hybridization, and binder‐free self‐supported arrays, is deployed to maximize the ECSA and minimize tortuous diffusion pathways (Scheme 3). While high‐surface‐area mesoporous and hollow structures excel at maximizing liquid‐solid contact, they often suffer from a gas‐trapping paradox where tortuous internal nanochannels trap evolved N_2_ and CO_2_ bubbles. This localized gas accumulation blocks active sites and triggers severe mass‐transport polarization at high current densities, effectively neutralizing intended structural advantages. Consequently, this dynamic loss of active surface area highlights a pervasive analytical flaw in structural engineering literature: the tendency to attribute bulk current density increases purely to enhanced intrinsic catalytic prowess. To accurately assess catalyst performance, rigorous normalization of anodic currents against the true, operando ECSA, rather than mere geometric area, is critical to decouple genuine intrinsic activity improvements from simple, yet often over‐estimated, surface‐area enlargement.

**SCHEME 3:**
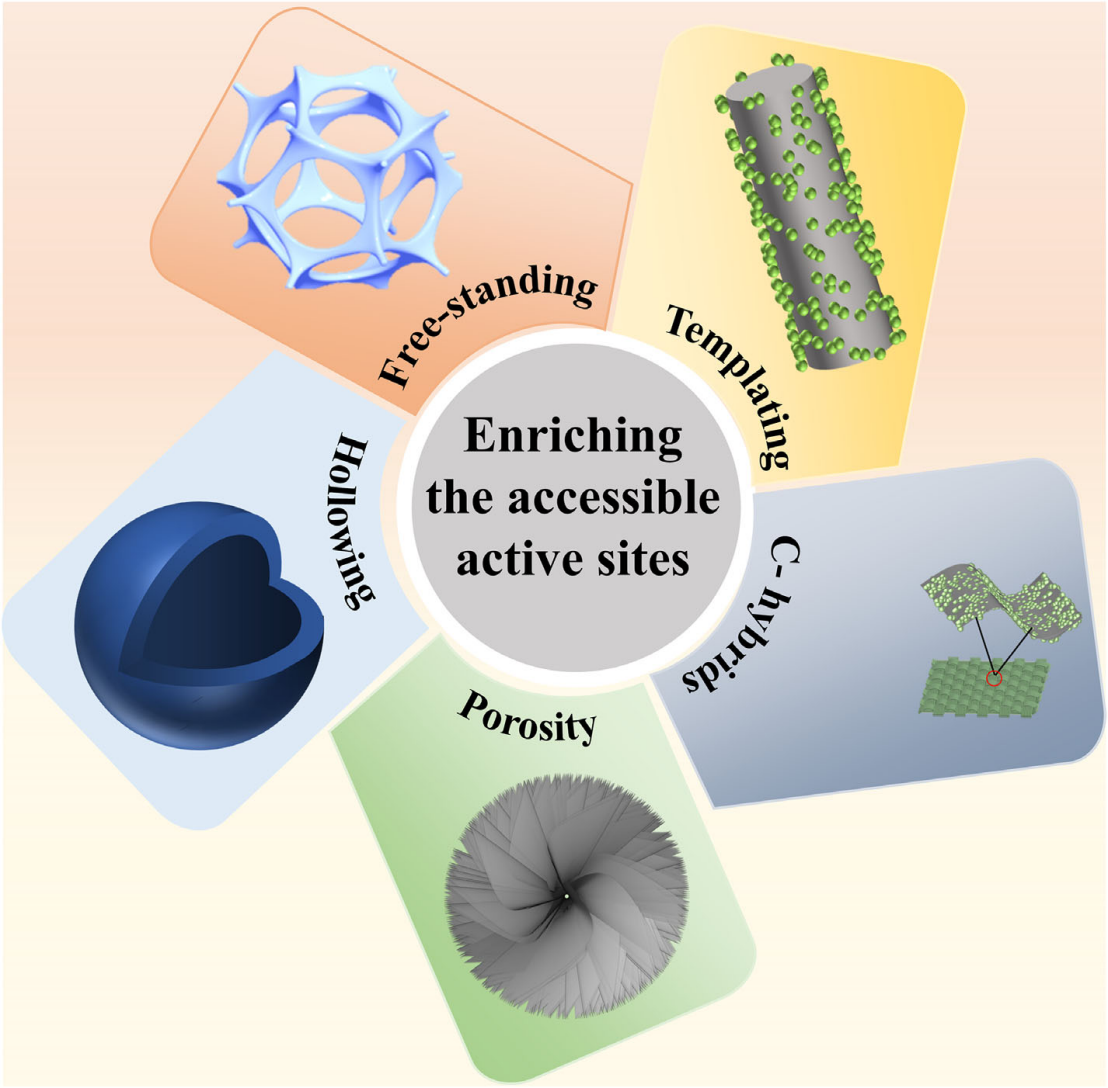
Schematic illustration of strategies for enriching accessible active sites in W‐based UOR electrocatalysts.

#### 3.2.1 Template‐Assisted Strategy

Template‐assisted synthesis provides a deterministic pathway for architecting highly defined, multi‐scale porous electrocatalysts. By employing transient sacrificial templates during high‐temperature processing, the anisotropic growth and severe thermal agglomeration of active phases can be strictly suppressed. For instance, the utilization of water‐soluble inorganic salts as transient spatial regulators dictates the assembly of two‐dimensional structural units into hierarchical, three‐dimensional frameworks (Figure 7a) [91]. Upon dissolution of the template, a dense network of interconnected meso‐ and macropores is exposed (Figure 7b). This deliberate architectural engineering resolves conflicting mass‐transport requirements: wide channels expedite the diffusion of bulky urea molecules and the rapid expulsion of gaseous products, while the high interfacial curvature maximizes the density of accessible catalytic nodes. Furthermore, the physical confinement imposed by the template during calcination stabilizes high‐valence heteroatoms within the lattice, enabling persistent electronic modulation of the catalytic centers (Figure 7c).

**FIGURE 7:**
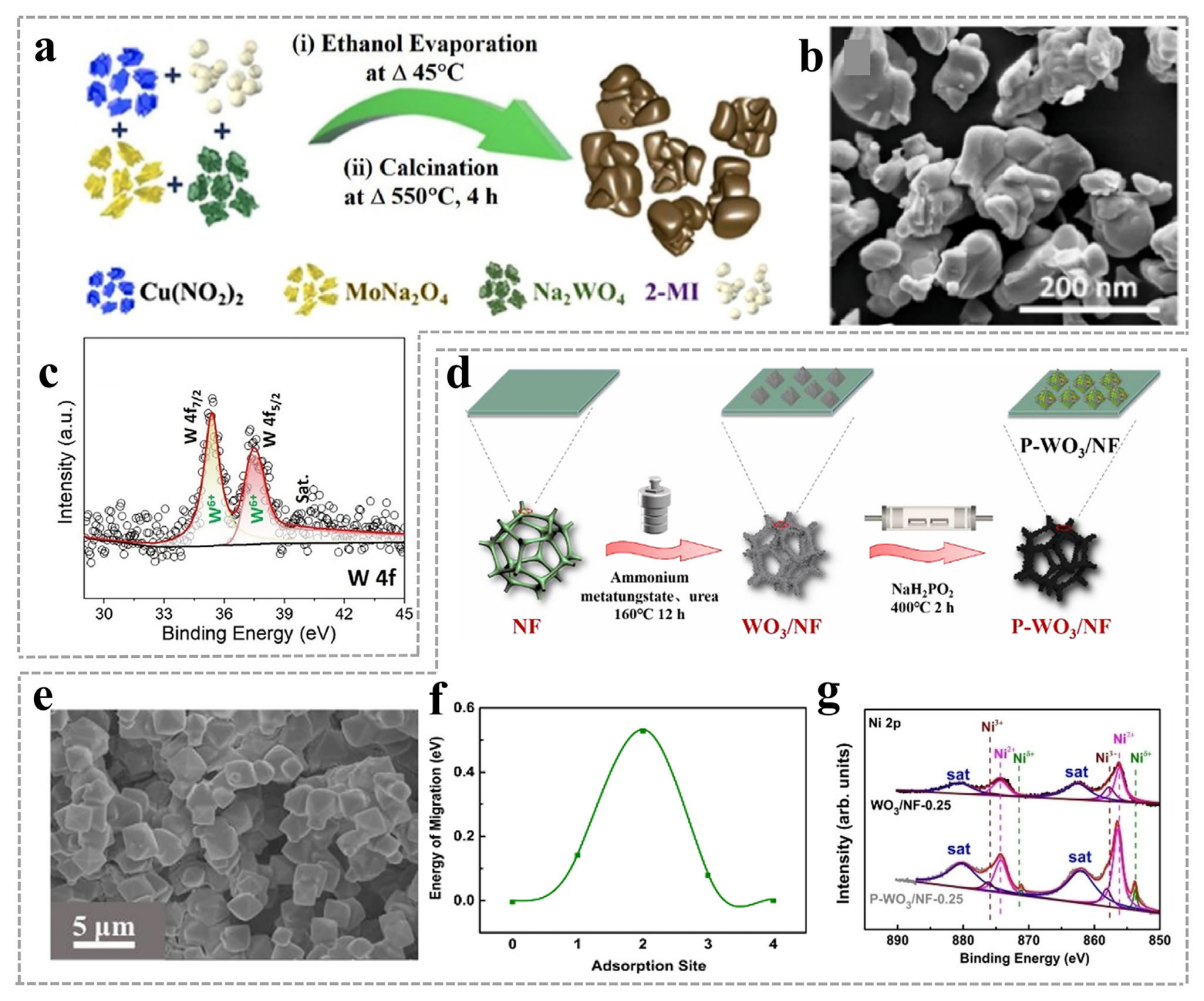
(a) Illustration depicting the synthesis process of W@Mo‐Cu MOF‐MOCC, (b) SEM for W@Mo‐Cu MOF‐MOCC, (d) XPS of W@Mo‐Cu MOF‐MOCC: W 4f. Reproduced with permission [91]. Copyright 2025, Elsevier. (d) The preparation of P‐WO_3_/NF, (e) SEM image of P‐WO_3_/NF‐0.25, (f) potential energy of Ni migration from W_16_O_48_ channel to another channel, (g) XPS spectra of WO_3_/NF‐0.25 and P‐WO_3_/NF‐0.25 for Ni 2p. Reproduced with permission [92]. Copyright 2022, Wiley VCH.

Beyond acting as mere spatial placeholders, templates can function as reactive scaffolds that direct solid‐state atomistic diffusion (Figure 7d). For example, rigid transition metal oxide frameworks, such as polyhedral WO_3_, provide well‐defined crystallographic channels that facilitate the directional infiltration of secondary catalytic metals like Ni (Figure 7e) [92]. Because the migration energy barrier within these pre‐defined lattice channels is fundamentally lower than bulk solid‐solution formation energies, secondary atoms can penetrate deeply into the core of template (Figure 7f). This confined infiltration generates spatially distributed, bi‐functional heterointerfaces, where the template core aggressively adsorbs urea molecules while the modified surface species drive the oxidative electron‐transfer cascade (Figure 7g).

Although template‐assisted synthesis excels at constructing highly accessible pore networks, it introduces several inherent vulnerabilities that strictly limit its practical viability. Fundamentally, the sacrificial nature of this approach severely compromises overall atom economy, establishing a structural reliance on complete template removal. Incomplete extraction often leaves behind trace residues, such as strongly adsorbing halide ions from salt precursors, which act as permanent poisons for electrophilic active sites and provoke severe localized galvanic corrosion under harsh anodic UOR potentials. Furthermore, while the resultant ultra‐thin pore walls dramatically amplify the initial electrochemically active surface area, they inherently lack long‐term mechanical resilience. During high‐current‐density operation, the intense hydrodynamic shear stress generated by the continuous nucleation and violent detachment of N_2_ and CO_2_ gas bubbles frequently induces catastrophic structural collapse and active mass shedding. To bridge the gap between idealized laboratory structures and scalable commercial deployment, future engineering models must pivot toward robust self‐templating precursors or fully recyclable scaffolding systems that circumvent both residue‐induced degradation and the poor atom efficiency of traditional hard templates.

#### 3.2.2 Carbon Nano‐Hybrids

Integrating W‐based active phases with highly graphitized carbon nano‐structures fundamentally transcends bulk electrical conductivity improvements; it institutes a robust spatial and electronic confinement mechanism. Carbon supports inherently dictate the geometric dispersion of active species while constructing continuous, low‐resistance charge‐transfer highways. In 2D confinement, matrices like reduced graphene oxide (rGO) supply expansive planar interfaces that physically restrict the nucleation and growth of metal tungstate nanoparticles [93]. This rigid spatial restriction directly suppresses thermal agglomeration and Ostwald ripening during synthesis (Figure 8a). Furthermore, the intimate physical coupling between the electroactive nanoparticles and the 2D sp^2^‐carbon lattice creates a highly conductive bridge (Figure 8b). This interface drastically mitigates charge transfer resistance, accelerates the cyclical regeneration of high‐valent metal centers, and fortifies the catalyst's resilience against intermediate poisoning (Figure 8c).

**FIGURE 8:**
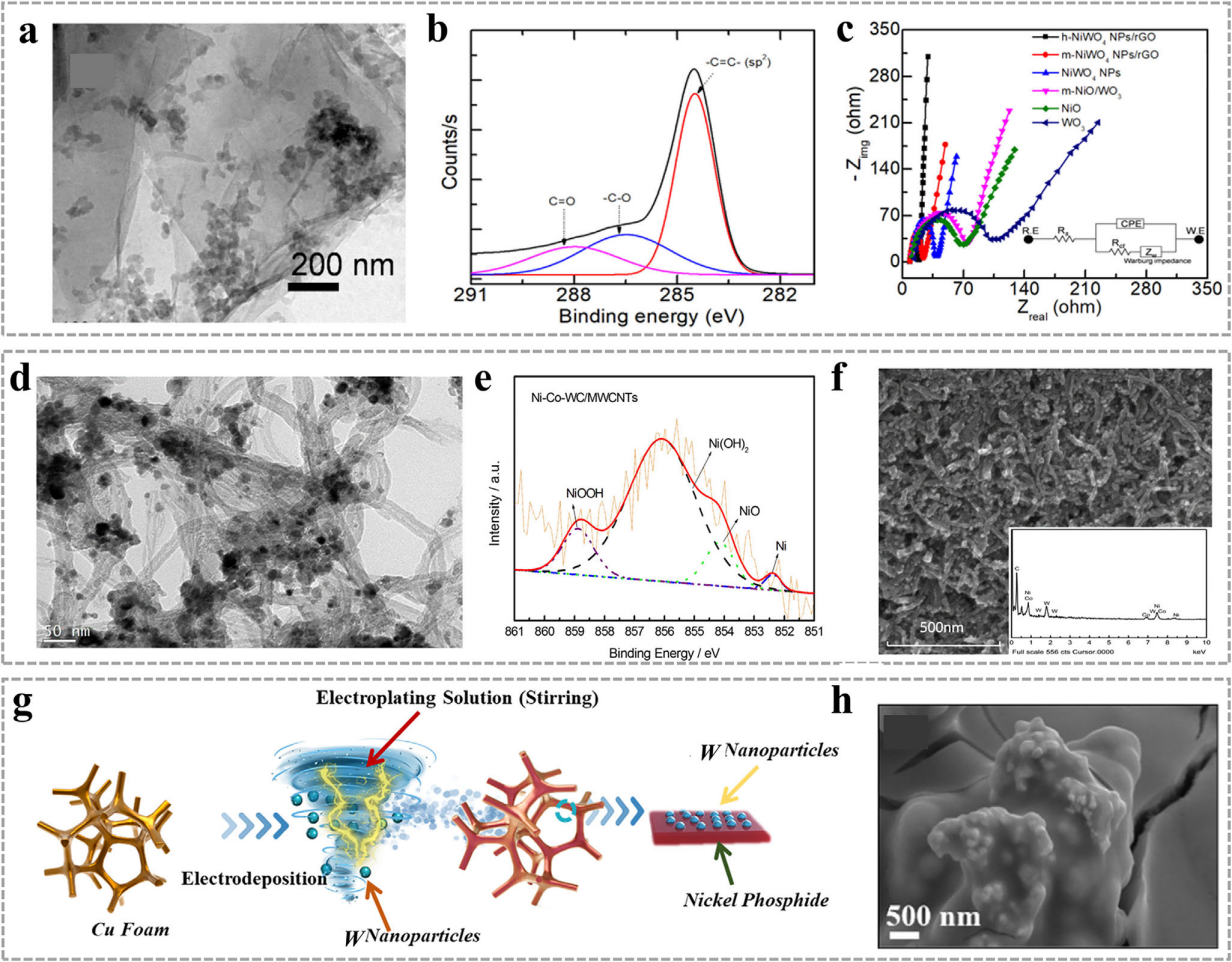
(a) TEM of h‐NiWO_4_ NPs/rGO, (b) high resolution C 1s XPS spectrum of h‐NiWO_4_ NPs/rGO composite, (c) Nyquist plots of different UOR catalysts. Reproduced with permission [93]. Copyright 2020, Elsevier. (d)TEM images of the Ni−Co−WC/MWCNTs, (e) XPS survey spectrums of Ni 2p spectrum for Ni–Co–WC/MWCNTs, (f)SEM of the Ni–Co–WC/MWCNTs catalyst (the inset is the corresponding EDX). Reproduced with permission [94]. Copyright 2018, American Chemical Society. (g) Schematic diagram for the synthesis of W/Ni‐P, (h) SEM image of W/Ni‐P. Reproduced with permission [95]. Copyright 2020, Elsevier.

Transitioning to 1D structures, matrices such as multi‐walled carbon nanotubes (MWCNTs) offer through‐hole nanochannels optimized for rapid, highly directional electron flux [94]. Anchoring transition metals and rigid carbides onto these 1D scaffolds generates a highly polarized ternary junction (Figure 8d). The robust electron extraction facilitated by the CNT backbone accelerates the crucial oxidation of Ni to active NiOOH phases, while the tethered carbide sites physically polarize the C‐N bonds within urea molecules (Figure 8e). Unlike dense 2D laminates that can occasionally impede vertical ion diffusion, the open 1D network ensures continuous, unobstructed mass transport of reactants and evolved gases to and from the confined active sites (Figure 8f). At the macroscopic 3D level, embedding tungsten components into highly porous, conductive metal‐carbon foams physically engineers extreme micro‐nano topographical roughness (Figure 8g) [95]. This 3D protrusion architecture geometrically amplifies the effective electrolyte‐electrode contact volume (Figure 8h). Beyond simple physical area expansion, the highly dispersed tungsten nodes operate as distal electronic modulators. They forcefully perturb the electron density of the surrounding transition metal matrix, significantly depressing the thermodynamic energy barrier required for active phase transition and orchestrating a holistic synergy between long‐range 3D mass transport and localized electronic activation.

Despite the structural and electronic advantages of carbon nano‐hybrids, a glaring vulnerability lies in the extreme susceptibility of defect‐rich carbon frameworks, such as rGO or functionalized MWCNTs, to catastrophic electrochemical oxidation under the highly anodic potentials required for UOR. This inevitable corrosion rapidly severs electrical pathways and triggers the shedding of active W/Ni phases. Furthermore, while electronic hybridization between the carbon support and d‐band metal/tungstate orbitals is frequently cited to explain enhanced activity, its exact nature is often merely hypothesized rather than empirically mapped. Therefore, rigorous operando spectroscopic studies are required to differentiate genuine synergistic orbital overlap from simple geometric dispersion effects, ensuring that observed performance gains are accurately understood and not masking underlying structural instability.

#### 3.2.3 Constructing Porous Structures

Constructing hierarchical porous structures fundamentally alters the electrocatalytic landscape of UOR by addressing both kinetic and mass‐transport limitations. A rigorously designed porous framework maximizes the ECSA while creating interconnected conduits for rapid liquid‐state reactant infusion and gaseous product expulsion. When active heterophases, such as transition metal sulfides or phosphides, are directly grown on conductive 3D substrates to form binder‐free, self‐supporting porous arrays, interfacial contact resistance is virtually eliminated (Figure 9a) [96]. This architectural continuum ensures seamless electron percolation. Furthermore, embedding distinct heterointerfaces within these porous networks catalyzes rapid interfacial charge transfer (Figure 9b). For instance, synergistic multi‐element doping within a porous framework not only structurally segregates active domains but also induces intense electron‐withdrawing effects (Figure 9c) [97]. This electronically starves the transition metal centers, accelerating their transition into high‐valent, catalytically active oxyhydroxide species (Figure 9d). From a mass‐transport perspective, specific porous topographies, like sea‐urchin‐like branched nanoneedle arrays, exert profound geometric effects (Figure 9e). The high surface curvature and abundant edge sites provide dense loci for urea activation. Crucially, these hyper‐branched structures frequently exhibit superaerophobic properties, ensuring that as gas bubbles nucleate, their contact area with electrode remains minimal. This facilitates ultra‐fast bubble detachment, thereby preventing the vapor‐lock effect and mass‐transfer polarization that typically paralyze dense planar electrodes at high current densities. By coupling these geometric advantages with nanoscale electronic modulation, carefully engineered porous arrays can sustain high current densities at reduced overpotentials (Figure 9f).

**FIGURE 9:**
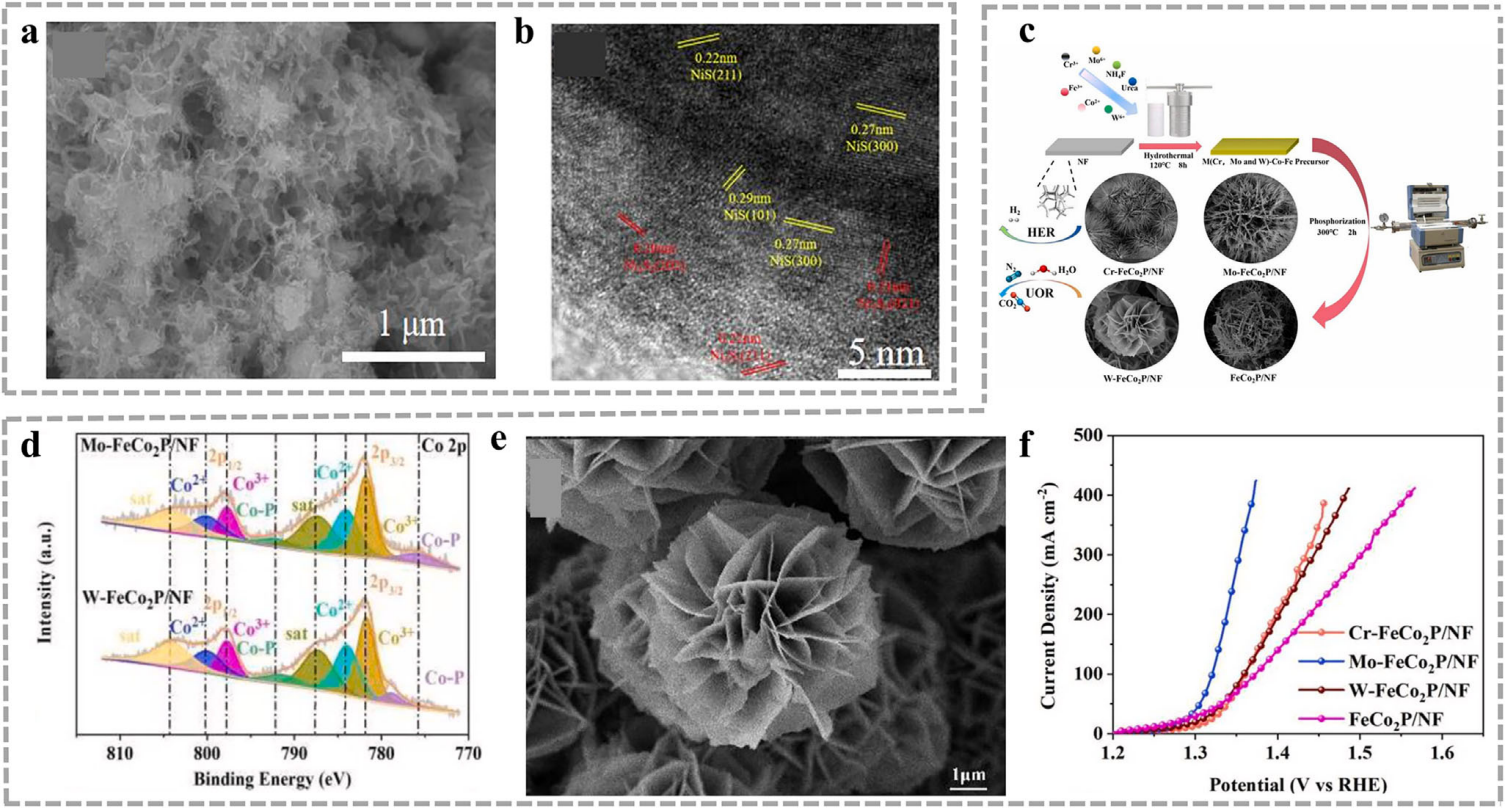
(a) SEM images of FeW‐Ni_3_S_2_/NiS@NF, (b) SAED images of FeW‐Ni_3_S_2_/NiS@NF. Reproduced with permission [96]. Copyright 2022, Elsevier. (c) Synthetic scheme of M‐FeCo_2_P/NF, (d) high‐resolution XPS spectroscopy of MoeFeCo_2_P/NF and W‐FeCo_2_P/NF in Co 2p, (e) SEM diagrams of W‐FeCo_2_P/NF, (f) UOR activity of different samples. Reproduced with permission [97]. Copyright 2022, Elsevier.

Despite the kinetic and mass‐transport advantages of hierarchical porous structures, their practical deployment is hindered by severe operational and scalable limitations. First, while macroscopic porosity facilitates bubble detachment, the intrinsically thin pore walls and high‐aspect‐ratio nanoneedles are mechanically fragile. Under the intense hydrodynamic shear forces generated by violent gas evolution during high‐current‐density UOR, these delicate structures are highly susceptible to mechanical fracturing, active mass shedding, and eventual catastrophic electrode failure. Compounding this physical vulnerability is the issue of internal concentration gradients; in deeply tortuous porous networks, rapid reactant consumption at the superficial layers frequently outpaces bulk diffusion. This localized depletion leaves the deep internal surface area electrochemically dormant, meaning the theoretical expansion of ECSA rarely translates into proportional high‐current performance. Finally, the synthesis of these binder‐free porous arrays relies heavily on intricate, high‐temperature hydrothermal and phosphidation/sulfidation protocols that are currently restricted to idealized, small‐scale metallic substrates like nickel foam. Scaling these highly sensitive batch processes to manufacture large‐area, uniform commercial electrolyzer stacks represents a profound engineering bottleneck that must be resolved to bridge the gap between laboratory architectural design and industrial application.

#### 3.2.4 Designing Hollow Structures

Hollow architectural engineering exploits expansive internal void spaces and ultra‐thin shell walls to maximize the exposure of catalytic interfaces. The foundational advantage of a hollow design is the dramatic amplification of surface‐to‐volume ratios, alongside an inherently high surface permeability. These geometric features drastically shorten radial diffusion lengths, allowing rapid mass transport of bulky urea molecules and accelerating the inward flux of electrolyte ions. Conversely, the porous exterior facilitates the efficient egress of gaseous byproducts, mitigating surface blockage. However, manipulating geometry in isolation is fundamentally insufficient for achieving high UOR turnover; it must be coupled with intrinsic electronic optimization. For example, fabricating elaborate hollow structures, such as MOF‐derived pinecone‐like spheres incorporating high‐valent tungsten (W^6+^), can yield immense BET specific surface areas (Figure 10a–c) [98]. Yet, if the incorporated W^6+^ nodes lack the necessary reversible redox pairs (unlike active Ni or Co species), they fail to synergistically modulate the adjacent active metal centers (Figure 10d). Without this critical electronic perturbation, the catalyst struggles to stabilize the essential high‐valence NiOOH active phase. In such scenarios, the geometrically abundant active sites remain electrochemically dormant, resulting in sluggish reaction kinetics and high operational overpotentials despite the theoretically advantageous hollow morphology.

**FIGURE 10:**
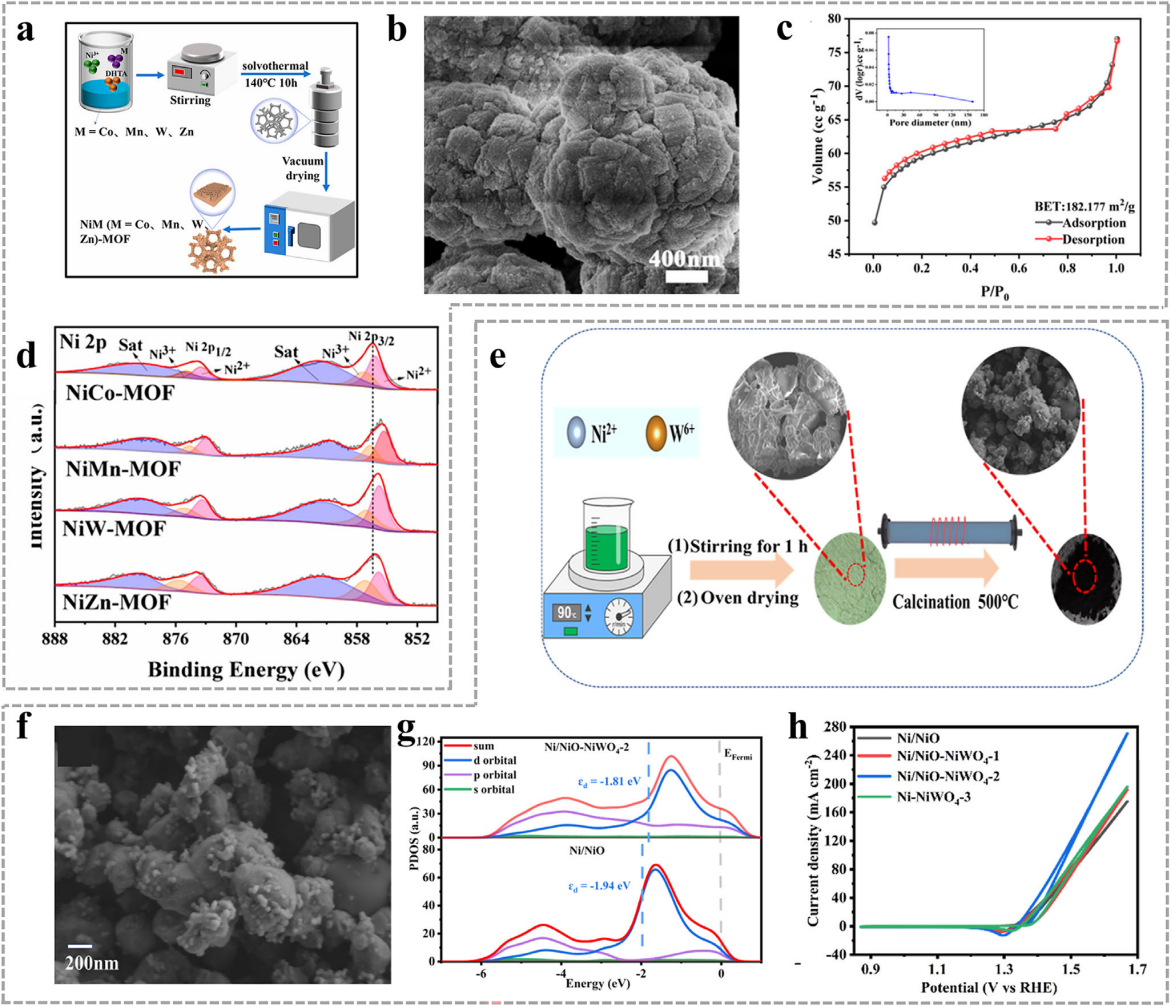
(a) Schematic diagram of the preparation process of NiM (M = Co, Mn, W, Zn)‐MOF, (b) SEM of NiW‐MOF, (c) N_2_ adsorption and desorption isotherm curve of NiW‐MOF, (d) XPS spectrum of NiM (M = Co, Mn, W, Zn)‐MOF: Ni 2p. Reproduced with permission [98], published by MDPI, 2025. Copyright 2025 by the authors. (d) Synthesis scheme of M‐FeCo_2_P/NF, (e) schematic synthesis diagram of sample, (f) SEM image of Ni/NiO‐NiWO_4_‐2 sample, (g) the adsorption comparison of different models for CO(NH_2_)_2_, CO_2_ and N, (h) CV curves of different samples in 1.0 M KOH+0.33 M urea. Reproduced with permission [99]. Copyright 2024, Elsevier.

To actualize the full potential of hollow constructs, spatial engineering must be synchronously integrated with effective electronic modulation. When functional dopants are successfully embedded within a hollow matrix, such as coupling NiWO_4_ into a hollow Ni/NiO shell via solvent evaporation, the advantages compound (Figure 10e,f) [99]. The hollow geometry physically alleviates liquid/gas mass‐transport limitations, while the active tungsten sites electronically redistribute charge. This electronic tuning optimizes the interaction between active sites and key UOR species by promoting urea adsorption and facilitating product desorption, while lowering the energy barrier of the rate‐determining urea activation step (Figure 10g). Consequently, the integration of targeted electronic reconstruction with hollow architectural design enhances the utilization of exposed active sites and promotes sustained UOR kinetics under high current densities. (Figure 10h).

Despite their theoretical advantages, hollow structural designs face severe operational and translational limitations. A pervasive flaw is the “dead volume” dilemma; if the mesoporous channels penetrating the outer shell are overly narrow or highly tortuous, the internal cavity remains inaccessible to the viscous alkaline electrolyte during *operando* conditions, meaning high BET surface areas frequently fail to translate into true ECSA. Furthermore, the ultra‐thin nature of these hollow shells renders them inherently fragile. Under the intense local pressure gradients induced by violent N_2_ and CO_2_ gas evolution at industrially relevant current densities, these delicate structures are highly susceptible to mechanical rupture and irreversible pulverization. Finally, these operational vulnerabilities are compounded by profound synthetic scalability constraints, as the fabrication of uniform hollow structures typically demands intricate, multi‐step templating, galvanic replacement, or Kirkendall effect pathways. The extreme sensitivity of these synthesis routes to kinetic parameters renders large‐scale, defect‐free commercial production highly prohibitive.

#### 3.2.5 Self‐Supported Catalytic Electrodes

The design of self‐supported catalytic electrodes fundamentally resolves the mass‐transport and electrical resistance limitations inherent to conventional slurry‐coated systems. By bypassing the use of non‐conductive polymer binders, which invariably encapsulate and deactivate a significant fraction of active sites, self‐supported geometries permit the direct integration of catalytically active phases onto 3D conductive current collectors, such as nickel foams or carbon cloth. This binder‐free monolithic design establishes a contiguous electron percolation network, ensuring rapid charge transfer directly from the electroactive boundary to external circuit, sustaining polarized local interfaces for efficient multi‐electron reactions.

The geometric and electronic advantages of self‐supported structures are profoundly amplified when coupled with spatial confinement and heteroatom modulation techniques like tungsten doping. In situ electrodeposition or carefully tuned hydrothermal growth processes allow for the construction of highly dense, geometrically defined nanostructures, such as distinct polyhedral nano‐arrays, anchored tightly to the macroscopic conductive scaffold (Figure 11a,b) [100]. The robust physical anchoring minimizes interfacial charge transfer resistance (Figure 11c), while the uniform geometric distribution inherently maximizes the ECSA. From an electronic perspective, the incorporated W atoms act as potent spatial modulators. Theoretical and experimental analyses demonstrate that W doping regulates the electronic structure of the Ni‐based matrix, enhancing the adsorption of reactive OH^−^ species and urea molecules at active sites. This electronic modulation promotes the formation of the high‐valence Ni^3+^ phase through facilitated OH^−^ adsorption and strengthens urea activation, thereby accelerating subsequent UOR processes (Figure 11d).

**FIGURE 11:**
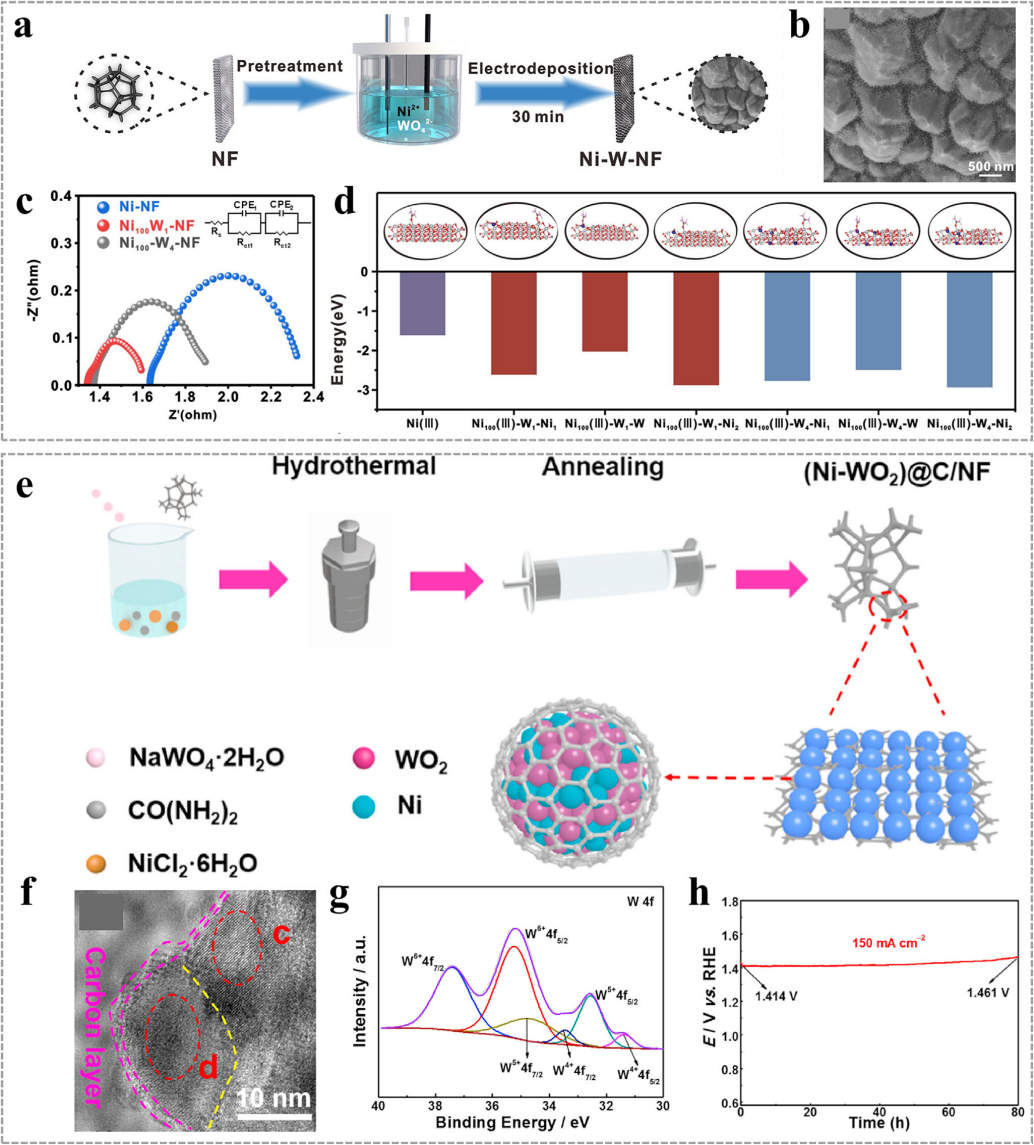
(a) Schematic illustration of Ni‐W deposition, (b) SEM images of Ni‐W‐NF, (c) Nyquist plots, (d) urea adsorption free energy at different sites. Reproduced with permission [100]. Copyright 2025, Elsevier. (e) Schematic diagram of the synthesis of (Ni‐WO_2_)@C/NF, (f) HRTEM images of (Ni‐WO_2_)@C/NF, (g) high‐resolution XPS spectra of W 4f, (h) the chronopotentiometry curve for (Ni‐WO_2_)@C/NF recorded. Reproduced with permission [101]. Copyright 2025, Elsevier.

Furthermore, integrating carbon encapsulation strategies within these monolithic structures significantly fortifies their anodic resilience [101]. Growing a conductive carbon sheath over W‐doped Ni arrays establishes a structurally robust core–shell interface that physically armors the metal‐oxide phases against the highly corrosive alkaline environment and the intense mechanical shear stress exerted by rapid gas evolution (Figure 11e,f). This protective yet conductive layer preserves the finely tuned W‐induced electronic states (Figure 11g), maintaining superior UOR kinetics and mitigating structural degradation during extended, high‐current‐density operation (Figure 11h).

Despite their mass‐transport and conductive advantages, self‐supported structures face severe operational and translational bottlenecks. A pervasive vulnerability is their reliance on 3D metallic substrates, such as nickel or copper foams, which are highly susceptible to irreversible oxidation and localized galvanic corrosion under the extreme anodic potentials mandated by UOR (Table 2). Over prolonged cycling, this deep substrate corrosion obliterates the underlying conductive network, leading to catastrophic active‐mass delamination. Compounding this structural fragility is the macroporous vapor‐lock effect; while the deeply tortuous internal voids of 3D foams offer vast geometric surface areas, they inherently trap the rapidly generated N_2_ and CO_2_ gases at industrially relevant current densities. This gas entrapment mechanically displaces the liquid electrolyte, starving deep active sites of urea reactants and rendering a significant portion of the theoretically accessible ECSA completely inert. Finally, translating these intricate structures from the laboratory to commercial deployment remains hindered by formidable manufacturing hurdles. The multi‐step in situ fabrication protocols, such as prolonged high‐temperature hydrothermal routines or precisely calibrated electrodeposition processes, are intrinsically batch‐dependent, making their integration into scalable, continuous roll‐to‐roll electrolyzer manufacturing a profound engineering challenge.

**TABLE 2:** Structural engineering strategies for W‐based UOR catalysts and their effects on active sites.

Catalysts	Major strategy	Structural feature	Active‐site effect	Refs.
W@Mo‐Cu MOF‐MOCC	Template‐assisted strategy	Hierarchical 3D meso	Increased active‐site accessibility; stabilized high‐valence sites	[91]
P‐WO_3_/NF‐0.25	Template‐assisted strategy	Directional metal infiltration and bifunctional heterointerfaces	Enhanced urea adsorption; electron transfer	[92]
M‐NiWO4 NPs/rGO	2D spatial confinement and conductive interfaces	2D spatial confinement and conductive interfaces	Accelerated high‐valence center regeneration; reduced poisoning	[93]
Ni−Co−WC/MWCNT	Carbon nano‐hybrids	1D nanochannels and polarized ternary junctions	Accelerated NiOOH formation; C‐N bond activation	[94]
W/Ni‐P	Carbon nano‐hybrids	3D porous protrusion architecture	Lowered active‐phase transition barrier; enhanced mass transport	[95]
FeW‐Ni_3_S_2_/NiS@NF	Constructing porous structures	Binder‐free 3D porous arrays with heterointerfaces	Enhanced charge transfer and active‐site accessibility	[96]
W‐FeCo_2_P/NF	Constructing porous structures	Sea‐urchin‐like branched nanoneedle arrays	Promoted high‐valent oxyhydroxide formation; urea activation	[97]
NiW‐MOF	Designing hollow structures	Hollow porous shells with high surface area	Increased active‐site exposure but limited NiOOH stabilization	[98]
Ni/NiO‐NiWO_4_‐2	Designing hollow structures	Hollow Ni/NiO shell with NiWO_4_	Optimized adsorption; lowered urea activation barrier	[99]
Ni_100_‐W_1_‐NF	Self‐supported catalytic electrodes	Anchored polyhedral nano‐arrays	Promoted Ni^3^^+^ formation; urea activation	[100]
(Ni‐WO_2_)@C/NF	Self‐supported catalytic electrodes	Conductive core–shell architecture	Preserved W‐induced electronic states; enhanced stability	[101]
NiWO_4_	Mesoporous structure engineering	Amorphous mesoporous framework with interconnected channels	Exposes active sites and facilitates NiOOH formation	[102]

Overall, active‐site enrichment strategies provide an important approach for improving the UOR performance of W‐based Ni catalysts by regulating the spatial architecture and interfacial transport characteristics of catalytic electrodes (Table 2). Although these structural engineering strategies focus on different aspects, they share the common objectives of increasing the number of electrochemically accessible active sites, facilitating reactant transport, and enhancing structural stability under operating conditions. Among them, template‐assisted strategies and hierarchical porous/hollow structures primarily construct open three‐dimensional channels to promote electrolyte penetration and gas release, whereas carbon nano‐hybrid structures further integrate highly conductive carbon frameworks to achieve synergistic optimization of electron transport and active‐phase dispersion. Self‐supported electrode structures, by minimizing the use of binders, establish direct electron‐transfer pathways and improve the structural integrity of catalytic electrodes. Overall, the essence of structural engineering is not simply to increase the surface area, but rather to achieve a synergistic optimization of active‐site accessibility, mass transport, and structural durability under realistic electrochemical conditions, providing a structural foundation for the development of efficient and robust W‐based UOR electrocatalytic systems.

## 4 Challenges

Although significant progress has been made in the study of W‐based catalysts for the UOR, several key challenges remain to be addressed before their practical application (Scheme 4):

**SCHEME 4:**
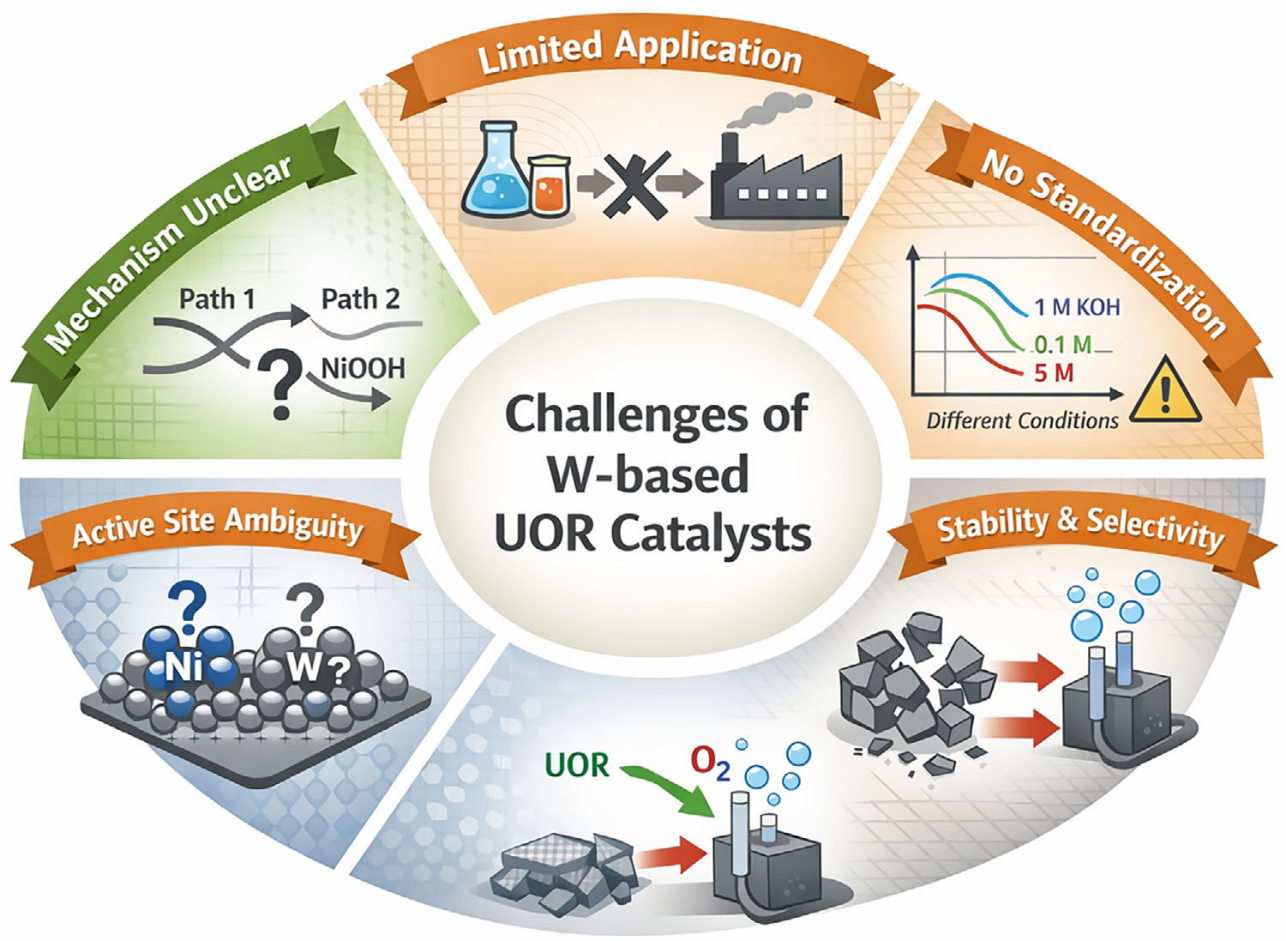
Schematic illustration of the key challenges for W‐based UOR catalysts.

(i) Limited understanding of the intrinsic catalytic mechanism. Currently, the understanding of the intrinsic catalytic mechanism of the UOR remains limited. Although possible reaction pathways such as the direct oxidation pathway and the indirect NiOOH‐mediated pathway have been proposed, the specific role of W in the UOR process is still not well understood. For instance, there is a lack of systematic knowledge regarding how the introduction of W modulates the electronic structure of the catalyst, and how these electronic structure changes subsequently influence the adsorption and transformation of reaction intermediates. Concurrently, regarding the adsorption and conversion mechanisms of key intermediates during UOR (e.g., *CO(NH_2_)_2_, *NH_2_), most conclusions are still primarily derived from DFT calculations, while direct experimental evidence from in situ/operando characterization techniques remains relatively scarce. Furthermore, during the actual electrocatalytic process, catalyst surfaces often undergo surface reconstruction, gradually evolving into new structures or active phases under reaction conditions. However, systematic theoretical and experimental investigations into the specific process of this dynamic structural evolution and its impact on UOR catalytic performance are still lacking. Therefore, an in‐depth elucidation of the structural evolution of W‐based catalysts during the reaction and its intrinsic correlation with the reaction pathway remains significant for a comprehensive understanding of their catalytic mechanism.

(ii) Ambiguity in identifying the real active sites. Most currently reported W‐based catalytic systems are typically composed of W species in conjunction with transition metals such as Ni and Co. When employed for the UOR under alkaline conditions, these materials often undergo varying degrees of structural evolution. This leads to significant uncertainty in the identification of the true active sites. Consequently, in these multi‐component systems, a definitive conclusion remains elusive as to whether W species function as active centers directly participating in the reaction, or primarily facilitate the reaction indirectly by modulating the electronic structure or through interfacial effects. Furthermore, because conventional characterization methods struggle to accurately track the dynamic changes of active sites under reaction conditions, direct evidence for the specific roles of different structural units on the catalyst surface during the reaction is still lacking. Therefore, developing an approach that combines in situ/*operando* characterization techniques with theoretical calculations is of great significance for identifying the true active sites and elucidating their synergistic mechanisms, thereby enabling a deeper understanding of the catalytic behavior of W‐based catalysts.

(iii) Stability and reaction selectivity in UOR. The structural stability of catalysts during the UOR and the issue of reaction selectivity remain significant challenges hindering their further application. Under practical electrocatalytic conditions, W‐based catalysts typically operate in strongly alkaline electrolytes at relatively high potentials for extended periods. This can lead to structural degradation, component migration, and other issues, thereby affecting their long‐term stability. Concurrently, these catalysts often undergo varying degrees of surface reconstruction during the reaction, with the original structure potentially transforming into new surface species or active phases. However, systematic research is still lacking regarding whether such dynamic structural changes can remain stable over the long term, as well as their specific impact on catalytic activity and durability. On the other hand, under alkaline electrolysis conditions, a competitive relationship generally exists between the UOR and the OER. As both reactions occur within a similar potential window, OER tends to proceed preferentially when the catalyst exhibits high activity for it, consequently reducing the Faradaic efficiency for UOR. Therefore, how to enhance the selectivity selectivity of catalyst for UOR and suppress the occurrence of OER, while simultaneously ensuring catalyst stability, remains a critical issue that urgently needs to be addressed in current research.

(iv) Lack of standardized evaluation protocols. The lack of a unified and standardized performance evaluation system is another issue in current UOR catalyst research that requires urgent attention. At present, electrochemical testing conditions often vary considerably across different studies, with parameters such as KOH electrolyte concentration, urea concentration, catalyst loading, and the type of reference electrode employed all differing. For example, regarding the definition of the substrate area, some studies calculate the current density based only on the single‐side geometric area of the electrode exposed to the electrolyte, while others include both sides. This discrepancy directly affects the calculated current density values, thereby further increasing the uncertainty when comparing performance across different studies. Such variations in experimental conditions can significantly influence the kinetics of the electrocatalytic reaction, leading to substantial differences in reported performance metrics like overpotential, current density, and kinetic parameters. Consequently, catalytic performances reported in different publications are often difficult to compare directly and objectively. Furthermore, some studies evaluate catalyst performance only within a three‐electrode setup, lacking systematic testing under conditions that more closely mimic practical applications. This, to some extent, limits an accurate assessment of the feasibility of the catalyst for practical applications. Therefore, establishing unified and standardized testing conditions and evaluation criteria, for instance clearly defining key parameters such as electrolyte composition, urea concentration, reference electrode type, and catalyst loading, is of great importance for achieving comparability between different research findings and for advancing the rational design and practical application of UOR catalysts.

(v) Limited progress toward practical applications. Although significant progress has been made in the fundamental catalytic performance of UOR, its translation toward practical applications remains limited. From an application perspective, UOR is generally considered to hold potential in several fields. However, different application scenarios each face their own key challenges. For instance, in UOR‐coupled hydrogen production systems, catalysts need to maintain long‐term stable operation at high current densities while exhibiting low working potentials and good durability. Currently, however, most studies are still conducted under lab‐scale testing conditions with small current densities. In the context of wastewater treatment applications, the majority of research is performed only in idealized alkaline electrolytes spiked with urea. Real wastewater systems, conversely, often contain various inorganic ions and organic impurities that can significantly impact catalyst activity and stability. Consequently, systematic investigations under conditions that more closely mimic realistic environments remain very limited. Furthermore, in the field of urea sensors, catalytic materials must not only possess good electrocatalytic activity but also simultaneously meet requirements for high sensitivity, a low detection limit, fast response, as well as good selectivity and anti‐interference capabilities, to achieve accurate detection of urea concentrations in complex environments. It is evident that advancing UOR catalysts from fundamental research toward practical application will still require more systematic and in‐depth studies focused on performance optimization, system validation, and adaptation to specific application scenarios.

## 5 Conclusions and Perspectives

The integration of tungsten into transition‐metal UOR electrocatalysts signifies a fundamental departure from purely geometric catalyst design toward precision electronic engineering. By leveraging tungsten's multivalent nature and electron‐withdrawing capability, researchers have modulated the d‐band centers of host matrices, regulated the binding strengths of key UOR intermediates, and partially overcome conventional thermodynamic scaling limitations. Coupled with advanced architectural motifs, ranging from hierarchical porosities to conductive carbon nano‐hybrids, these hybridized systems have achieved unprecedented intrinsic activities and mass‐transport efficiencies.

Despite these kinetic triumphs, the field stands at a critical translational juncture. To bridge the gap between idealized laboratory demonstrations and industrial viability, future research must aggressively pivot from empirical trial‐and‐error toward deterministic, operando‐guided design. Moving forward, the following strategic imperatives must dictate the trajectory of UOR electrocatalysis:

(i) Relying on static theoretical models is no longer sufficient. The deployment of multimodal *operando* spectroscopies, tightly coupled with AIMD incorporating explicit solvation, is imperative. This synergy will finally capture the transient structural reconstruction of W‐doped active phases and the true identity of catalytic intermediates under intense anodic polarization.

(ii) The dual challenge of *operando* stability and competitive selectivity represents the next frontier of material synthesis. Catalytic structures must be explicitly engineered to resist catastrophic heteroatom leaching and bulk substrate oxidation, while simultaneously maintaining a strict thermodynamic bias against the parasitic OER to ensure near‐unity Faradaic efficiency.

(iii) The establishment of stringent, universal evaluation protocols is an absolute prerequisite for authentic benchmarking. The community must standardize critical testing parameters, mandate ECSA‐normalized kinetic reporting, and enforce in situ gaseous product quantification to eliminate the pervasive reporting of morphological artifacts as intrinsic activity gains.

(iv) The path of UOR research must align with the harsh conditions of practical deployment. Future explorations should prioritize the scalable fabrication of complex, low‐cost assemblies, such as high‐entropy alloys and fully binder‐free MEA structures. Crucially, these systems must be validated under brutal, non‐idealized conditions, including real‐world wastewater matrices containing competitive poisoning ions, while sustaining commercial current densities.

Ultimately, realizing the dual promise of UOR, simultaneous environmental remediation and energy‐efficient hydrogen generation, relies on transcending current fundamental benchmarks. By strictly adhering to these rigorous, application‐driven patterns, W‐based electrocatalysts can effectively transition from theoretical marvels to foundational components of sustainable energy infrastructure.

## Author Contributions

Y. Zeng and Y Wang performed investigation, writing – original draft, review & editing. S. Donkor, D. Cairns, and X. Zheng performed literature scoping, visualization, writing – review & editing. S. Lu, Q. Hua, and B. B.Xu performed conceptualization, resources, project administration, writing – review and editing, and supervision.

## Conflicts of Interest

The authors declare no conflicts of interest.

## Data Availability

No primary research results, software or code have been included, and no new data were generated or analyzed as part of this review. The copyright was acquired and attached in the Supporting Information.
